# Exploring the chemical components of Kuanchang-Shu granule and its protective effects of postoperative ileus in rats by regulating AKT/HSP90AA1/eNOS pathway

**DOI:** 10.1186/s13020-024-00892-3

**Published:** 2024-02-21

**Authors:** Wen-Qian Duan, Ming-Chen Cai, Qi-Qi Ma, Peng Huang, Jia-Hui Zhang, Tian-Fu Wei, Dong Shang, Ai-Jing Leng, Jia-Lin Qu

**Affiliations:** 1 Clinical Laboratory of Integrative Medicine, The First Affiliated Hospital of Dalian Medical University, No. 222, Zhongshan Road, Dalian, 116011 China; 2 Institute (College) of Integrative Medicine, Dalian Medical University, No. 9, South Road of Lvshun, Dalian, 116044 China; 3 Department of Traditional Chinese Medicine, The First Affiliated Hospital of Dalian Medical University, No. 222, Zhongshan Road, Dalian, 116011 China

**Keywords:** Kuanchang-Shu granule, Postoperative ileus, Chemical profile, Network pharmacology analysis, Molecular docking, AKT/HSP90AA1/eNOS

## Abstract

**Background:**

Postoperative ileus (POI) is a common obstruction of intestinal content passage caused by almost all abdominal operations that seriously strokes the quality of life of patients. Kuanchang-Shu granule (KCSG), a classic modified prescription based on “Da-Cheng-Qi Decoction”, has obtained satisfactory efficacy in the clinical therapeutics of POI. However, its material basis and holistic molecular mechanism against POI have not been revealed.

**Methods:**

The chemical ingredients of KCSG were first characterized by ultra-performance liquid chromatography coupled with quadrupole time-of-flight mass spectrometry (UHPLC-QTOF-MS). Subsequently, an integration strategy of the network pharmacology and molecular docking based on above identified ingredients was performed to unveil the potential targets involved in the treatment of KCSG on POI. Finally, intestinal manipulation induced rat POI model was constructed to verify the efficacy and predicted mechanism of KCSG against POI.

**Results:**

In total, 246 ingredients mainly including organic acids, flavonoids, quinones, alkaloids, terpenoids, phenylpropanoids and phenols were identified. 41 essential ingredients, 24 crucial targets as well as 15 relevant signaling pathways were acquired based on network pharmacology analysis. Pharmacodynamic research showed that KCSG treatment could protect intestinal histological damage, promote the recovery of measurement of gastrointestinal transit disorder and inhibit the secretion of myeloperoxidase in the distal ileum tissues. The up-regulated expression of p-AKT and down-regulated expression of p-eNOS and HSP9OAA1 predicted by molecular docking and validated by western blotting showed that AKT/eNOS/HSP90AA1 pathway may be one of the crucial mechanisms that mediates the protective effect of KCSG.

**Supplementary Information:**

The online version contains supplementary material available at 10.1186/s13020-024-00892-3.

## Introduction

Postoperative ileus (POI), an obstruction of intestinal content passage, is a common gastrointestinal surgery disease. It is estimated that as high as 40% of patients suffered from POI after laparotomy [1]. Among which, gastrointestinal, cardiothoracic and orthopedic surgery occupied the top three places successively, the former even account for 24% [2, 3]. In recent years, the occurrence of POI has been reduced at a certain extent accompanied by the improved accuracy of early diagnosis on POI. Moreover, the prognosis of patients was improved by perioperative management that focuses on early rehabilitation [4]. However, surgical treatments of POI often cause serious complications such as intestinal fistula, and conservative treatments including fasting and continuous gastrointestinal decompression treatment usually show therapeutic effects slowly. Therefore, effective intervention measures and drugs are desperately needed to reverse or block the occurrence and development of this disease.

It is noteworthy that a large number of effective schemes are provided for clinical treatment along with vigorous progression research of combination of traditional Chinese medicine and western medicine [5]. Several formulas, single herb or active ingredient of traditional Chinese medicines, such as Shenhuang plaster [6], Zingiberis Siccatum Rhizoma [7], *Cistanche deserticola* [8] and hesperidin [9], had played unique advantages in the treatment of gastrointestinal diseases, whose mechanisms were related to inhibiting inflammatory responses by regulating ghrelin and other intestinal hormones, activating the transient receptor potential A1 (TRPA1) channels in entero-chromaffin cells, stimulating interstitial Cajal cells and stimulating Ca^2+^-dependent myosin phosphorylation. Among them, Da-Cheng-Qi Decoction (DCQD) is a representative laxative prescription with remarkable effect recorded in “Shang Han Lun”, which can effectively improve the inflammatory symptoms of intestinal peristalsis and obstruction, alleviate gastrointestinal paralysis, and improve gastrointestinal motor function with a rapid and highly potent effect [10, 11]. However, DCQD is mainly used for treating the syndrome of accumulated heat in large intestine with a rapid and highly potent effect. Most weak patients who could not bear such potent efficacy after abdominal surgery and long-term fasting need other alternative medicine.

Kuanchang-Shu granule (KCSG) is an improved in-hospital preparation based on DCQD, by adding the herbs with regulating qi and activating blood circulation, combining reinforcement and elimination, moistening the intestines and freeing the stool. It is composed of ten herbs, namely Rhei Radix et Rhizome (RRR), *Magnolia officinalis* Cortex (MOC), Raphani Semen (RS), Aurantii Fructus Immaturus (AFI), Codonopsis Radix (CR), Scrophulariae Radix (SR), Persicae Semen (PS), Angelicae Sinensis Radix (ASR), Aucklandiae Radix (AR) and Salviae Miltiorrhizae Radix et Rhizoma (SMR). Among them, RRR, MOC, RS and AFI are Monarch herbs in KCSG and known as eliminating heat, purging fire and promoting qi circulation to alleviate middle energizer. CR, SR, PS and ASR are Minister herbs in KCSG and known as invigorating spleen, replenishing qi, promoting fluid production and blood circulation. Also, AR is the assistant herb in KCSG and known as promoting qi circulation to alleviate middle energizer. SMR is the guide herb in KCSG and known as promoting blood circulation and dispersing blood stasis. At present, KCSG has achieved good clinical efficacy in the treatment of gastrointestinal motility disorder, inflammatory intestinal obstruction and habitual constipation after abdominal surgery. However, the complexity of the ingredients in the KCSG makes it impossible to determine which ingredients make them effective and how they work.

**Fig.1:**
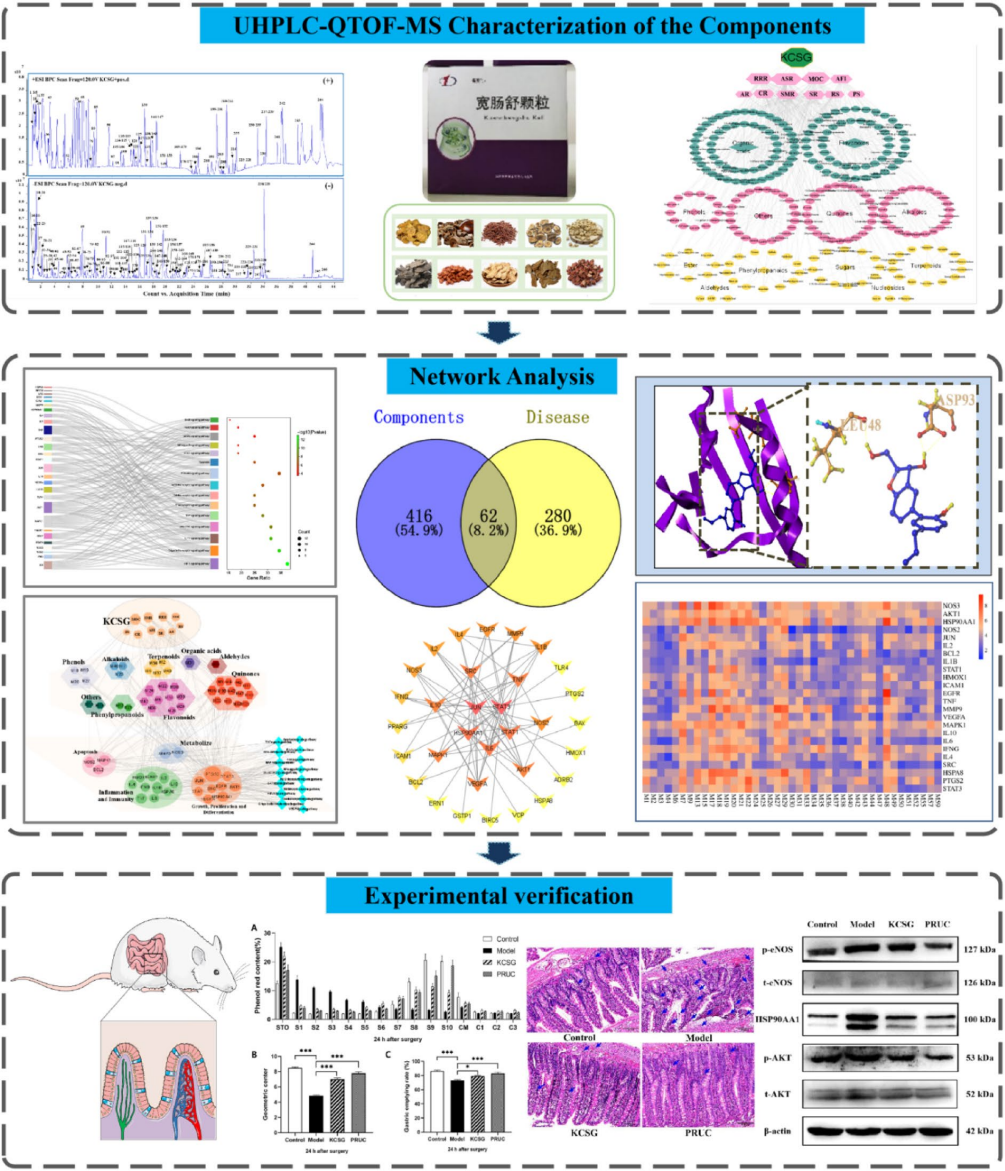
The schematic diagram of the present study

Systems pharmacology is a subject stemming from the systems biology, which aims at finding out the synergistic relationship between multi-targets and multi-compounds by analyzing biological networks and screening the nodes which are of specific interest [12]. Over the past decades, it has been successfully used for elucidating the mechanism of compound preparations in vivo [13]. For example, Jia and Wang et al. provided new insights for infection mechanism and potential targets of several Chinese patent medicines and formulas against coronavirus disease 2019 (COVID-19) [14, 15]. Therefore, this study drew lessons from the research ideas of systems pharmacology. First of all, an ultra-performance liquid chromatography coupled with quadrupole time-of-flight mass spectrometry (UHPLC-QTOF-MS) technology was used to exploring the chemical components of KCSG. Subsequently, network pharmacology and molecular docking were carried out to explore its core components and key targets in our study. In addition, an intestinal manipulation (IM)- induced POI rat model was established to evaluate efficacy and mechanism of KCSG against POI, which provides research basis for further experimental studies and clinical applications (Fig. 1).

## Materials and methods

### Chemicals and materials

Formic acid of MS grade was obtained from Fisher Scientific Company Inc. (FairLawn, NJ). HPLC–MS grade acetonitrile and methanol were supplied by Merck Company Inc. (Darmstadt, Germany). Ultra-pure water (18.2 MΩ) was purified using the Milli-Q water purification system (Millipore, Milford, MA, USA). All other grades of analytic chemicals were provided by Tianjin Concorde Technology Co., Ltd. (Tianjin, China).

Sinapine (**13**), sinapine thiocyanate (**45**), amygdalin (**67**), honokiol (**70**), ferulic acid (**90**), tanshinone I (**98**), chrysophanol (**169**), magnolol (**175**), costunolide (**179**), emodin (**191**), tanshinone IIA (**211**) and synephrine (**243**) were supplied by the National Institutes for Food and Drug Control (Beijing, China). The purity of each reference exceeded 98%.

KCSG was provided by the First Affiliated Hospital of Dalian Medical University (Liaoning, China). Prucalopride was purchased from Jiangsu Haosen Pharmaceutical Co., Ltd. (Jiangsu, China). Anti-Akt and phospho-Akt antibodies were purchased from Proteintech (1:1500, Hubei, China). Anti-eNOS and phospho-eNOS antibodies were purchased from Affinity Biosciences (1:500, Jiangsu, China). Anti-HSP90AA1 antibody was purchased from Abcam (1:1000, Cambridge, MA, USA). HRP Goat Anti-Rabbit IgG (H + L) was purchased from ABclonal Technology Co., Ltd. (1:5000, Hubei, China). BCA Protein Assay Kit and Total Protein Extraction kit were obtained from KeyGEN Biotech (Jiangsu, China). MPO detection kit was obtained from Nanjing Jiancheng Bioengineering Institute (Jiangsu, China).

### UHPLC-QTOF-MS/MS condition analysis

The condition of chromatographic separation and mass detection was consistent with the method mentioned previously [16, 17].

### Data mining for network pharmacology

The available ingredients contained in the KCSG prescription were obtained after identifying by UHPLC-QTOF-MS/MS, which were screened out through the Lipinski’s rule of five [18] and entered into the Molispiration Smiles database (https://www.molinspiration.com/cgi-bin/properties) [17]. Then, the Canonical SMILES structure formats of these compounds were supplemented through the PubChem (https://www.ncbi.nlm.nih.gov/pubmed/) [19], and the targets of active component were obtained from the Swiss Target Prediction Database (http://www.swisstargetprediction.ch/) [20] by inputting the Canonical SMILES structure formats. In addition, the names of the constituents were also converted into the Traditional Chinese Medicine Systems Pharmacy Database and Analysis Platform (TCMSP) (http://LSP.nwu.edu.cn/tcmsp.php) [21] to obtain more target information. Finally, the names of proteins were entered into official gene symbols (Homo sapiens) by using the UniProt Knowledgebase (https://www.uniprot.org/) [22].

The targets for POI were obtained from the Omim database (https://www.omim.org/), Etdbase database (http://ctdbase.org/voc.go?type=disease), Gene Cards database (http://www.genecards.org/) [23] and DisGeNET database (http://www.disgenet.org/web/DisGeNET/menu) [24]. Protein–Protein Interaction (PPI) with a persuasive score greater than 0.95 was analyzed and selected with the STRING database (https://www.string-db.org/) [25] to ensure the accuracy of the results.

To clarify the relationship between the signaling pathways and key targets, KEGG pathway enrichment was founded in the Database for Annotation, Visualization and Integrated Discovery (DAVID) (https://david.ncifcrf.gov/home.jsp) [26].

Then, the four networks were constructed by exploiting the network visualization software Cytoscape 3.9.1 [19], which can be used for integrating, analyzing, and visualizing the complex network analysis. The networks were constructed as follows: (1) constituents-putative targets network of KCSG; (2) disease targets network of POI; (3) active ingredients-intersection target PPIs-pathways network; (4) herb-compound-pathways-targets network analysis. In the network plots, components, targets, and pathways were indicated by nodes, and all the edges were all represented by the interactivity of different nodes. The connected edges number was shown as a node “degree”.

### Molecular docking

The molecular-docking module in SYBYL-X 2.0 [27] was applied to predicted active ingredients with the selected POI targets. Above all, the 2D structure of the constituent was attained from the PubChem database (https://www.ncbi.nlm.nih.gov/pubmed/) [28] in SDF format. Next, the SDF format was converted into the 3D structure in mol2 format for the preparation of micromolecule ligand compounds by SYBYL-X 2.0 software. Subsequently, PDB ID of targets was searched by the uniport database (http://www.uniprot.org/), [17], and we got the protein crystal structure of key targets from the RCSB Protein Data Bank database (http://www.rcsb.org) [29]. Before the docking process, a series of treatments were carried out to prepare the macromolecular receptor target proteins, including adding polar hydrogen atoms, taking out original ligands and water molecules, renovating amino acids, and building binding pockets. The surflex-dock total score was given by SYBYL-X 2.0 software to express the affinities of binding. The higher score was represented as the stronger interaction between bioactive compounds and proteins.

### Postoperative ileus (POI) model and sample collection

Adult male Sprague–Dawley (SD) rats (8 weeks, 250 ± 25 g) acquired from the Laboratory Animal Center of Dalian Medical University were randomly divided into 4 groups: control (CON) group, POI group, KCSG group and Prucalopride (PRUC) group (n = 10), and maintained in regular cages with the environment under controlled temperature at 22 ± 2 °C, 55 ± 5% humidity and a 12 h light cycles condition. All rats were acclimatized the condition with free access to water and solid food for 1 week prior to experiments. The animal experiments were approved by the Ethics Committee and Animal Experiment Committee of Dalian Medical College (Permit ID: AEE22042).

As described in the previous reports, the experimental animals were subjected to standardized IM with slight modifications [30]. The manipulation was performed under sterile conditions as follows. Briefly, the experimental rats were subjected to a laparotomy under anesthesia by inhaling isoflurane. Subsequently, the small bowel was carefully exteriorized and placed on the moist gauze. Next, the entire region of the small bowel was manipulated back and forth for 5 min with a sterile cotton swab. After the manipulation, the abdomen was kept open with the bowel covered with sterilized gauze for an additional 10 min. Then, the viscera were placed back into the abdomen. After laparotomy, the rat’s abdomen was closed with interrupted sterile non-absorbable surgical sutures, and the rats recovered quickly from surgery in a heated (32 °C) cage with free to drink but not food. The rats that received laparotomy alone without intestinal manipulation were indicated as the corresponding controls.

Tsuchida et al.'s study showed that the period of POI in animal experiments usually lasting for 24 h [31, 32], combined preoperative and postoperative drug administration were adopted to have a dual effect of prevention and treatment. Chinese herbal formulas, single herbs or monomers are often administered for 3–4 days before surgery and 1–2 times after surgery in treating POI [7, 33–36]. Similarly, 5-hydroxytryptamine receptor 4a (5-HT4R) agonists such as prucalopride was often used as western medicine therapy in the treatment of POI [31, 37], which are often administered once before surgery and 2–3 times after surgery. Accordingly, the rats were orally administrated with KCSG solution (13.5 g/kg/day) for four days and PRUC solution (2 mg/kg/once, positive drug) for 3 times during the experiment. The rats in the control and model group took the same dose of normal saline as KCSG and PRUC.

At 24 h after IM, part of the intestine tissues was immediately collected and fixed with 4% paraformaldehyde (*w/v*) for hematoxylin and eosin (H&E) staining. The others were harvested and frozen at − 80 °C for gastrointestinal transit, MPO activity and western blot analysis.

### Histopathological observation

The distal ileum fixed with 4% paraformaldehyde was dehydrated, embedded in paraffin, and sliced into 2 µm thickness. Subsequently, the staining of resultant sections was conducted with H&E reagent. And the histopathological changes were photographed by an optical microscope (Olympus, Japan). Finally, an ameliorative histopathologic score according to Cuzzocrea [38] was used to assess the trauma of intestinal mucosa of the randomized and unlabeled specimen.

### Measurement of Gastrointestinal Transit (GIT)

With some slight modification to the method of Tsuchida [31], GIT was measured by the geometric center (GC) calculated according to the weighted average distribution of phenol red (n = 10) as follows. After 22.5 h of IM, rats were orally administered with 0.2 mL of phenol red solution. After 90 min, the stomach and cecum were separated into two single segments (STO and CM), the intestine was divided into ten segments (S1-S10), and the colon was cut into three segments (C1-C3). The tissues of gut segments were minced and homogenized in the solutions of 10 mL of NaOH (0.1 M) with the mixing of vortex. Next, the mixture was kept at room temperature for 1 h and centrifugation at 3000×*g* for 10 min. The trichloroacetic acid was added to the supernatant to remove the precipitates of proteins. After centrifugation again (10,000×*g*, 20 min), 0.1 mL of the supernatant and 0.133 mL of 0.5 M NaOH were combined to read the optical densities at 560 nm by an enzyme-labeled instrument.

The calculation of the gastric emptying (GE) and GC of distribution of phenol red was performed as reported previously [39, 40].

### Detection of myeloperoxidase (MPO) level in intestinal tissues

MPO activity was used as a reliable index for indicating the accumulation of leukocytes and neutrophils. After thawed and transferred onto a filter to remove the liquid, the wet weight of the frozen intestinal muscular layer was determined. Subsequently, 100 mg of the distal ileum was homogenized and fluidized with the reagent to obtain 10% homogenate. O-dianisidine was used as peroxidase substrates. The absorbance of the muscular solution was measured by the changes at 460 nm with a 96-well plate reader.

### Western blot analysis

Intestinal tissue proteins in different groups were collected with Total Protein Extraction kit. Concentrations of proteins were quantified by bicinchoninic acid method. 40 μg proteins were resolved by SDS-PAGE and transferred onto PVDF membranes. After blocking with a blocking buffer containing 5% fresh non-fat milk, the membranes were applied with primary antibodies (eNOS, AKT, HSP90AA1) overnight and HRP-conjugated secondary antibody for 2 h. The blots were normalized by β-actin and visualized by the Tanon-5200 Imaging System (Tanon, Shanghai, China). Eventually, Image J was used to quantitatively analyze the probed bands.

### Statistical analysis

Quantitative data were presented as means ± standard deviation of the mean (SEM) and performed with GraphPad Prism 8.0.1 software (San Diego, CA, USA) from independent experiments. Results were carried out by one-way analysis of variance (ANOVA) test and post hoc Tukey’s test. A probability value (P) of *P* < 0.05 was considered to be statistically significant.

## Results

### UHPLC-QTOF-MS characterization of the components in KCSG

**Fig. 2:**
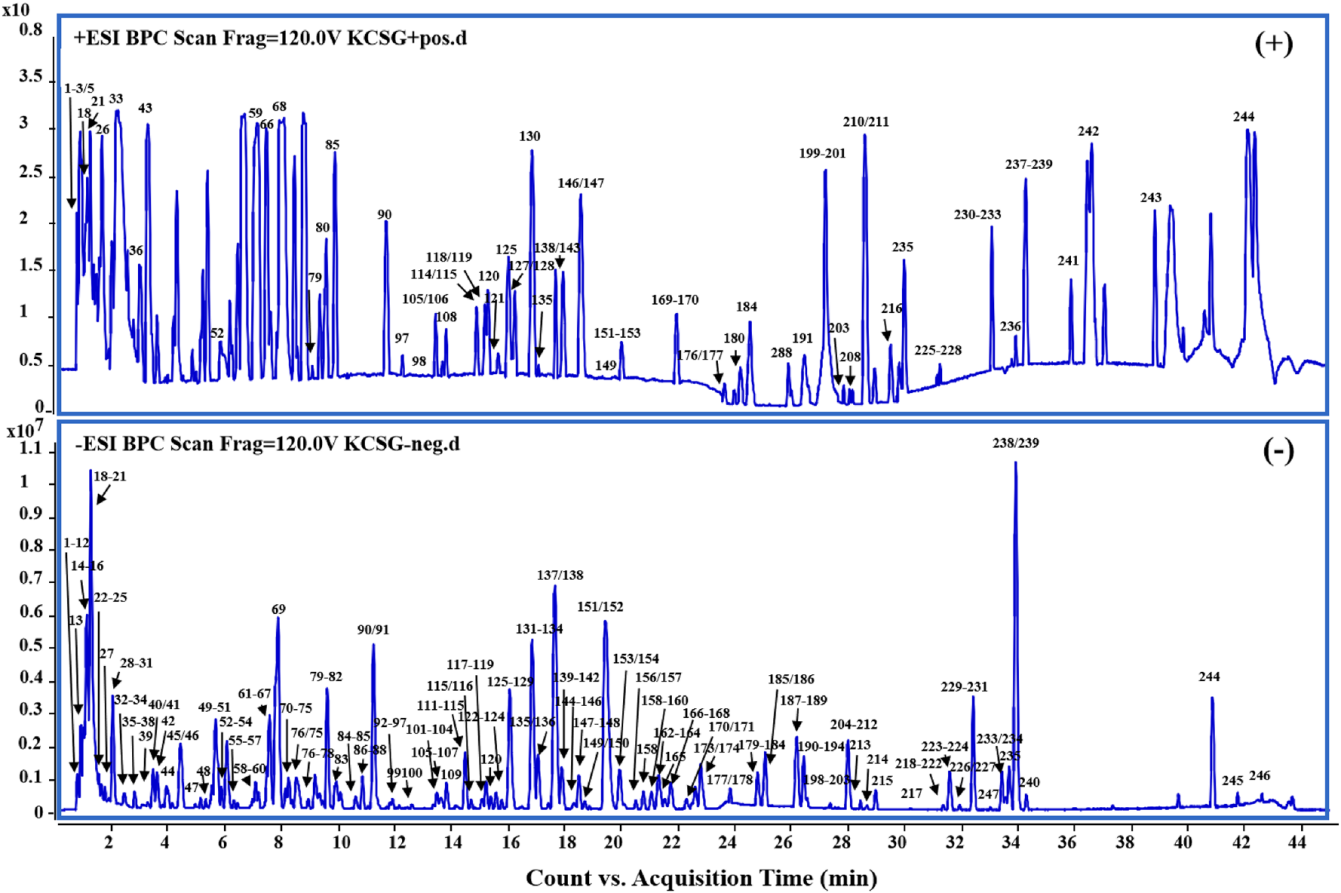
Representative base peak chromatogram (BPC) of KCSG in the positive and negative ions mode, respectively

According to the constituent database of KCSG constructed and data appraisal software, a total of 246 constituents including 58 organic acids, 51 flavonoids, 25 quinones, 23 alkaloids, 14 terpenoids, 13 phenylpropanoids, 13 phenols, 12 sugars, 8 esters, 3 nucleosides, 3 aldehydes and 23 miscellaneous compounds were tentatively characterized under the optimized conditions (Fig. 2, Additional file 1: Table S1).

Among them, 12 compounds were unambiguously identified as sinapine (**13**), sinapine thiocyanate (**45**), amygdalin (**67**), honokiol (**70**), ferulic acid (**90**), tanshinone I (**98**), chrysophanol (**169**), magnolol (**175**), costunolide (**179**), emodin (**191**), tanshinone IIA (**201**) and synephrine (**243**) by direct comparison of the retention time and quasi-molecular ions. While high-accurate quasi-molecular ions such as [M + H]^+^, [M + Na]^+^, [M − H]^−^, [M + Cl]^−^ and [M + HCOO]^−^ were used to identify the other ingredients within a mass error of 10.0 ppm. Information regarding the 246 compounds is outlined in Additional file 1: Table S1.

### Network construction and analysis

#### Target network construction of KCSG components and postoperative ileus

**Fig. 3:**
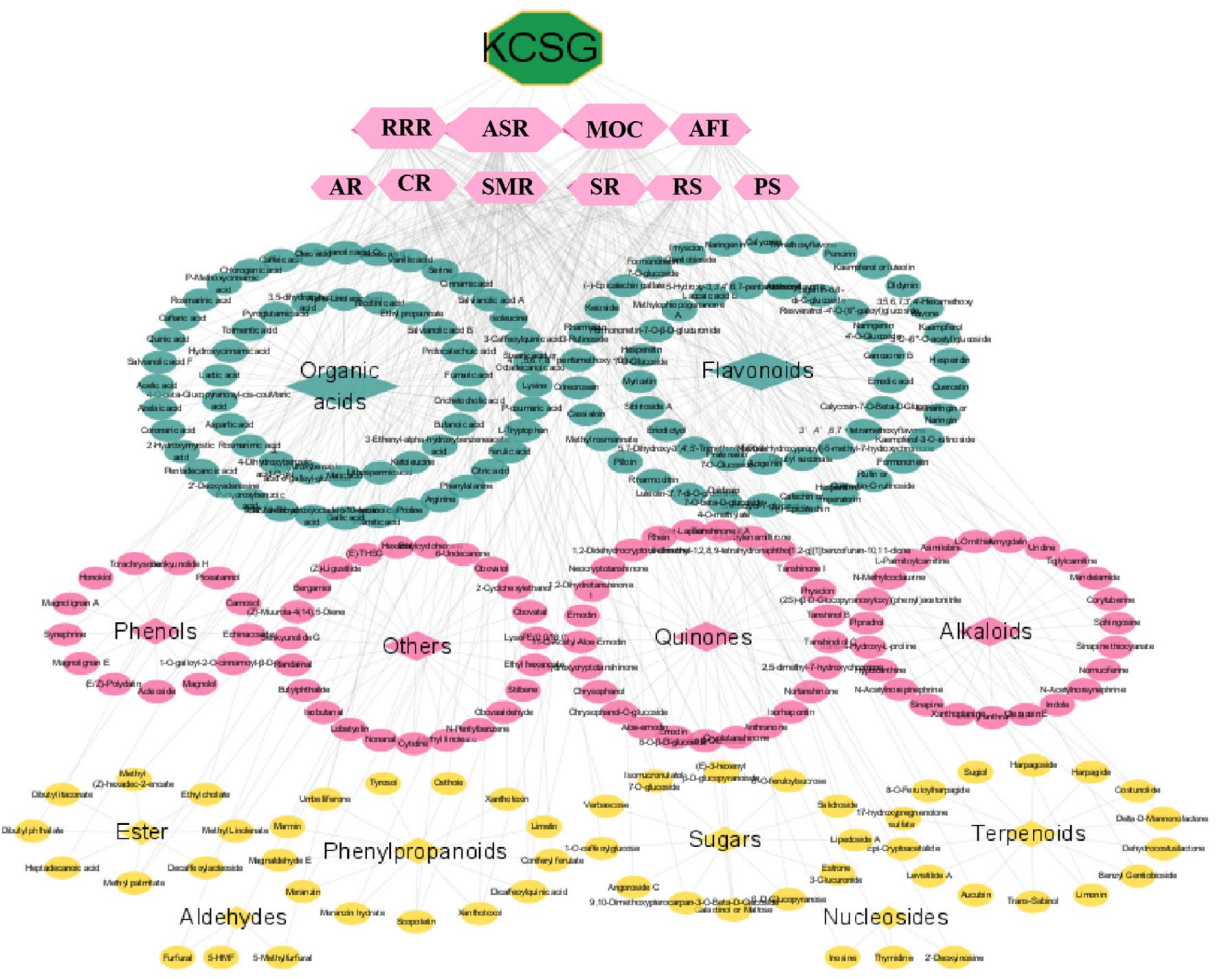
Prescription-herb-compound-category network of KCSG. (The green octagons and pink hexagons represent KCSG and herbs, respectively. Green, pink and yellow nodes represent compounds. Green, pink and yellow diamonds represent category. The size of nodes is in proportion to their degree)

**Fig. 4:**
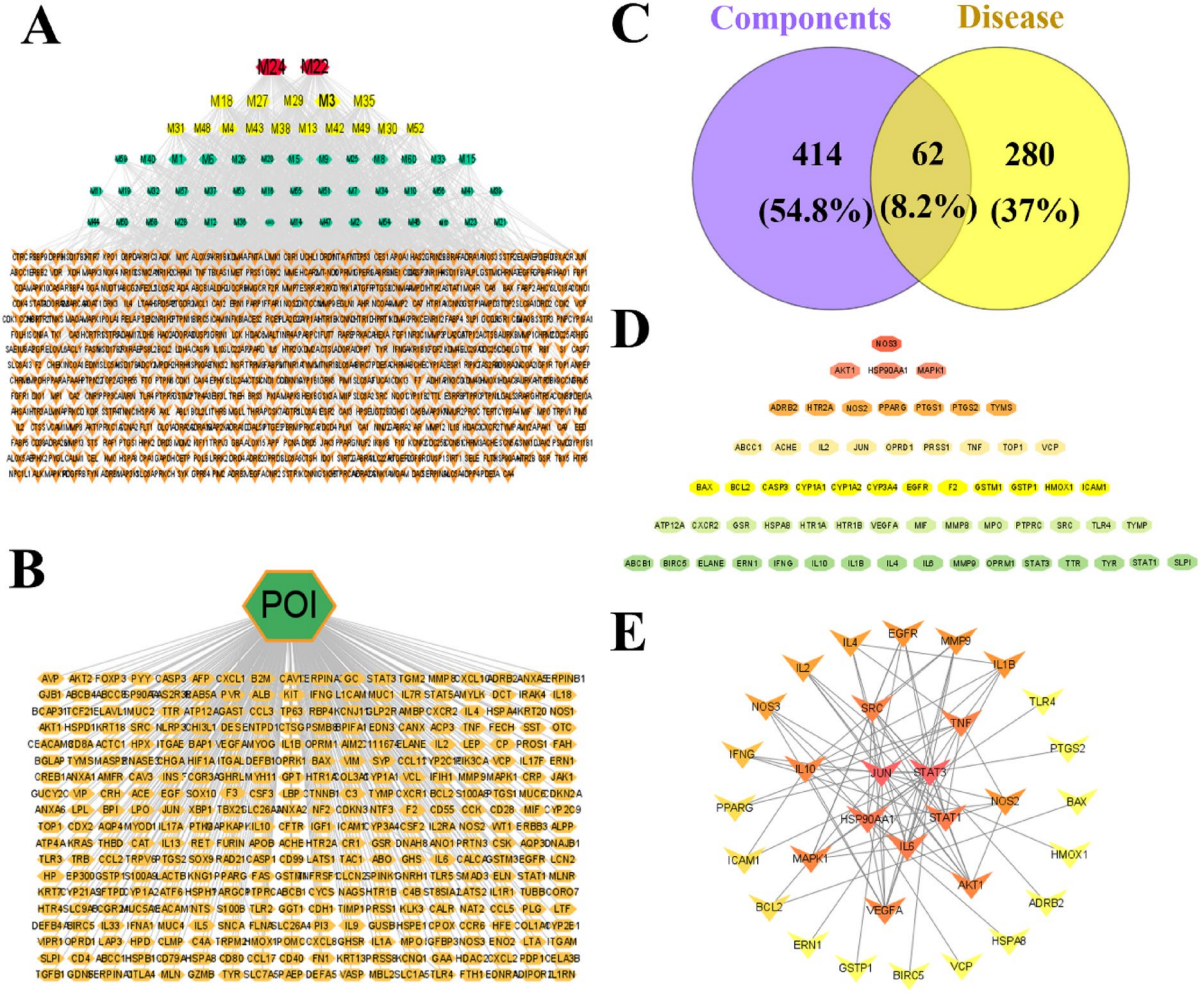
The active ingredients-target interactions for KCSG against POI. **A** The network of connections between active ingredients and targets. The red, yellow, and green nodes represent ingredients in KCSG, while the orange ones represent targets. **B** Disease-target networks of POI. The green nodes represent POI while the yellow ones represent targets. **C** The Venn diagram for targets of ingredients and the disease. The purple circle is the KCSG target genes, the yellow circle is the POI target genes. The gray area is the 62 overlap targets. **D** 62 overlap targets. **E** PPI network of the KCSG ingredients targets against POI. Network nodes represent proteins, and edges represent the interaction between proteins. The color from yellow/orange/red indicated that the redder the rectangle is, the more important the node is in the PPI network

Prescription-herb-compound-category network of KCSG constructed by Cytoscape was shown in Fig. 3. From this network, RRR and MOC were considered to be the main botanical source of 246 constituents, while flavonoids and alkaloids were considered to be the main botanical category due to their higher degree. According to the Lipinski's rule and the value of OB and DL, a total of 60 active compounds were screened out to focus on more important information (Additional file 2: Table S2). Combined with the chemical profile of KCSG and database search results, 476 targets associated were predicted as potential targets of KCSG and shown in Fig. 4A. Similarly, 342 targets of POI obtained from GeneCards and DisGeNET databases were collected (Fig. 4B).

#### Analysis of PPIs

To analyze the characteristics of KCSG in the treatment of POI, the component-targets and disease-targets were intersected by Cytoscape 3.9.1 software and 62 common targets were acquired (Fig. 4C, D). A protein interaction network of the above-mentioned 62 targets screened out was built by the STRING database. According to the confidence score > 0.95 of network construction, 32 key targets were further screened and considered as candidate targets (Fig. 4E).

#### Metabolic pathway analysis and network construction

**Fig. 5:**
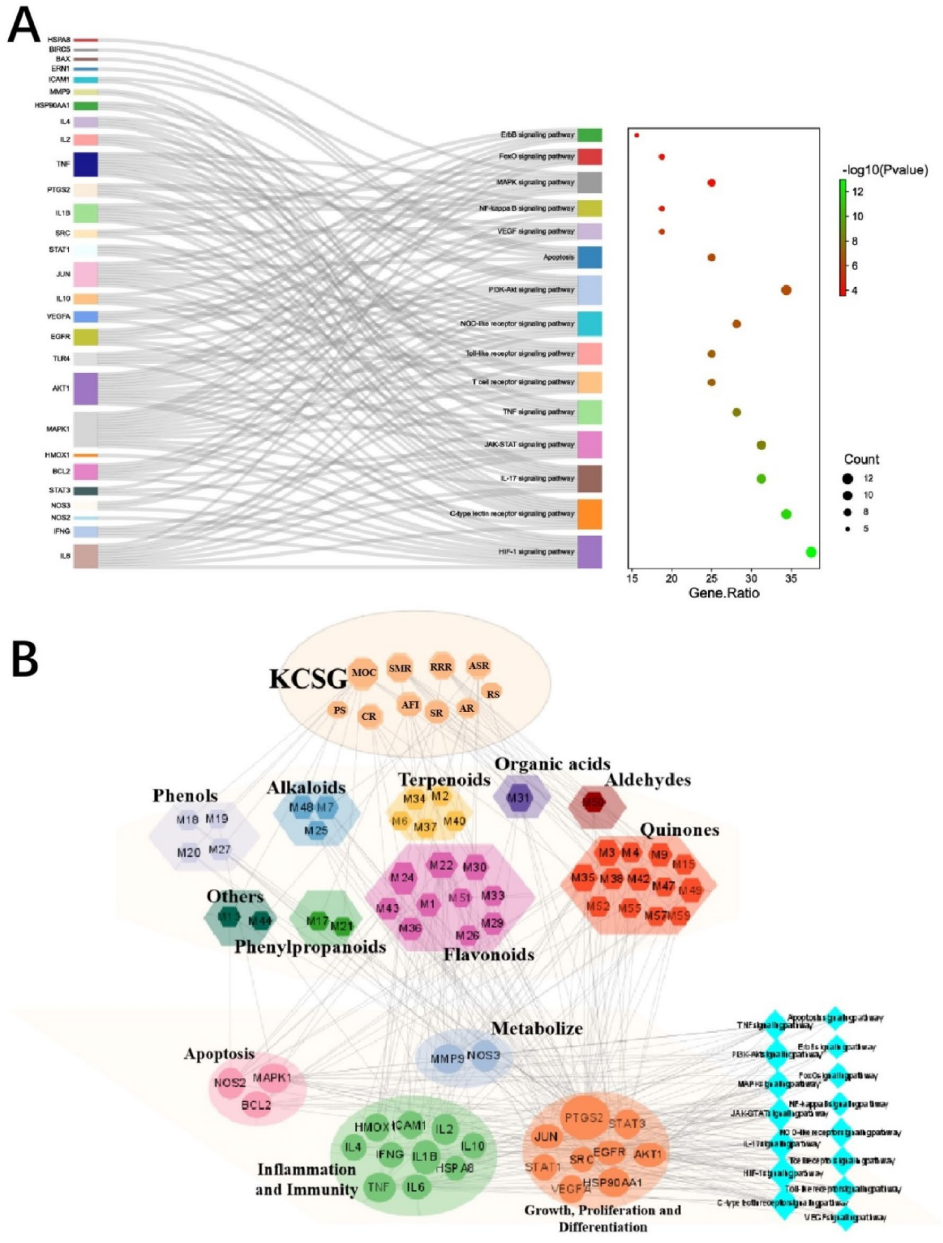
**A** The top 15 signaling pathways of KEGG pathway enrichment analysis for the KCSG in treatment of POI. **B** The relationship between the core targets with the active ingredients of KCSG and pathways, and the distribution of the KCSG in the POI network. The orange octagons stand for the composition of KCSG's herb, and the pentagon stand for the active ingredient in KCSG. The blue diamond represents the related pathways, and the ellipse nodes represent target genes. The size of nodes is in proportion to their degree

**Fig. 6:**
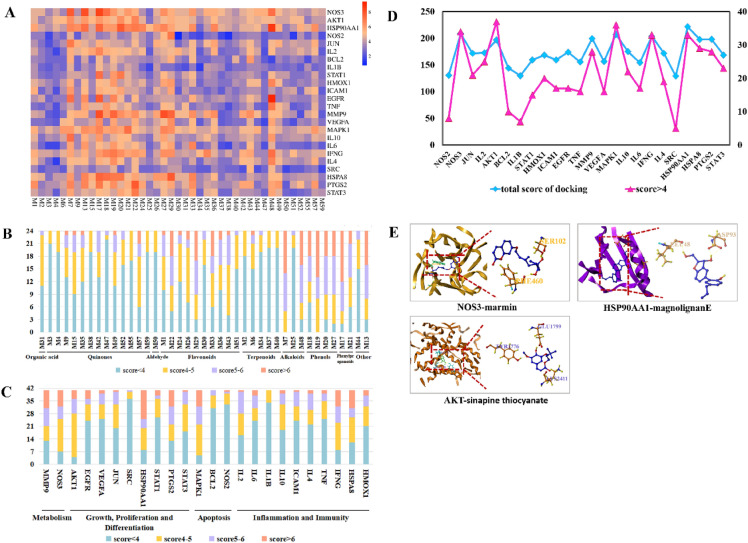
Virtual docking of the binding of key compounds with related proteins was analyzed by SYBYL-X 2.0 software. **A** Heat map of docking score; **B**–**D** component and target docking score; **E** molecular docking analysis of representative combinations

KEGG pathway enrichment analyses of the intersecting targets were carried out with the DAVID website to obtain relational pathways. As a result, 111 pathways were acquired with statistically significant (*p* < 0.05, except cancer pathways). After removing irrelevant disease pathways, the first 15 pathways were screened (Fig. 5), and the 24 targets contained in the pathways were considered as the core targets. 41 active constituents associated with the 24 core targets were predicted as key active constituents (Additional file 3: Table S3, Additional file 4: Table S4). Accordingly, key active constituents-core targets-pathway network of KCSG was shown in Fig. 6.

### Molecular docking

RCSB Protein Data Bank was applied to collect a crystal structure of the target, which aims to investigate the relationship between core ingredients and key targets. Subsequently, the docking relationship was analyzed with SYBYL-X 2.0 and more critical targets were obtained with the screening value (score > 5.0) (Fig. 6A and B). As a result, eNOS (PDB ID: 4d1p), AKT (PDB ID: 5wby) and HSP90AA1 (PDB ID: 5j80) exposed better binding ability with alkaloids, coumarins, phenols and flavonoids constituents, respectively. Moreover, magnolignan E (from MOC), marmin (from AFI), and sinapine thiocyanate (from RS) revealed higher binding activity (Fig. 6C and D). The representative binding conformations (NOS3-marmin, HSP90AA1-magnolignan E, and AKT-sinapine thiocyanate) were shown in Fig. 6E.

As shown in Fig. 6E, two hydrogen bonds were identified between NOS3 and marmin (**193**), which are the interaction with phenylalanine (PHE460) and serine (SER102) respectively. Similarly, the three interactions consist in the combination of HSP90AA1 and Magnolia E (**198**), two of which are the interaction with aspartic acid (ASP93) and the other one interacted with leucine (LEU48). In addition, three hydrogen bonds were found between AKT and sinapine thiocyanate (**45**), which interacted with tyrosine (TYR1776), glutamic acid (GLU1799) and lysine (LYS2411), respectively.

### Experimental evaluation

#### Histological evaluation

**Fig. 7:**
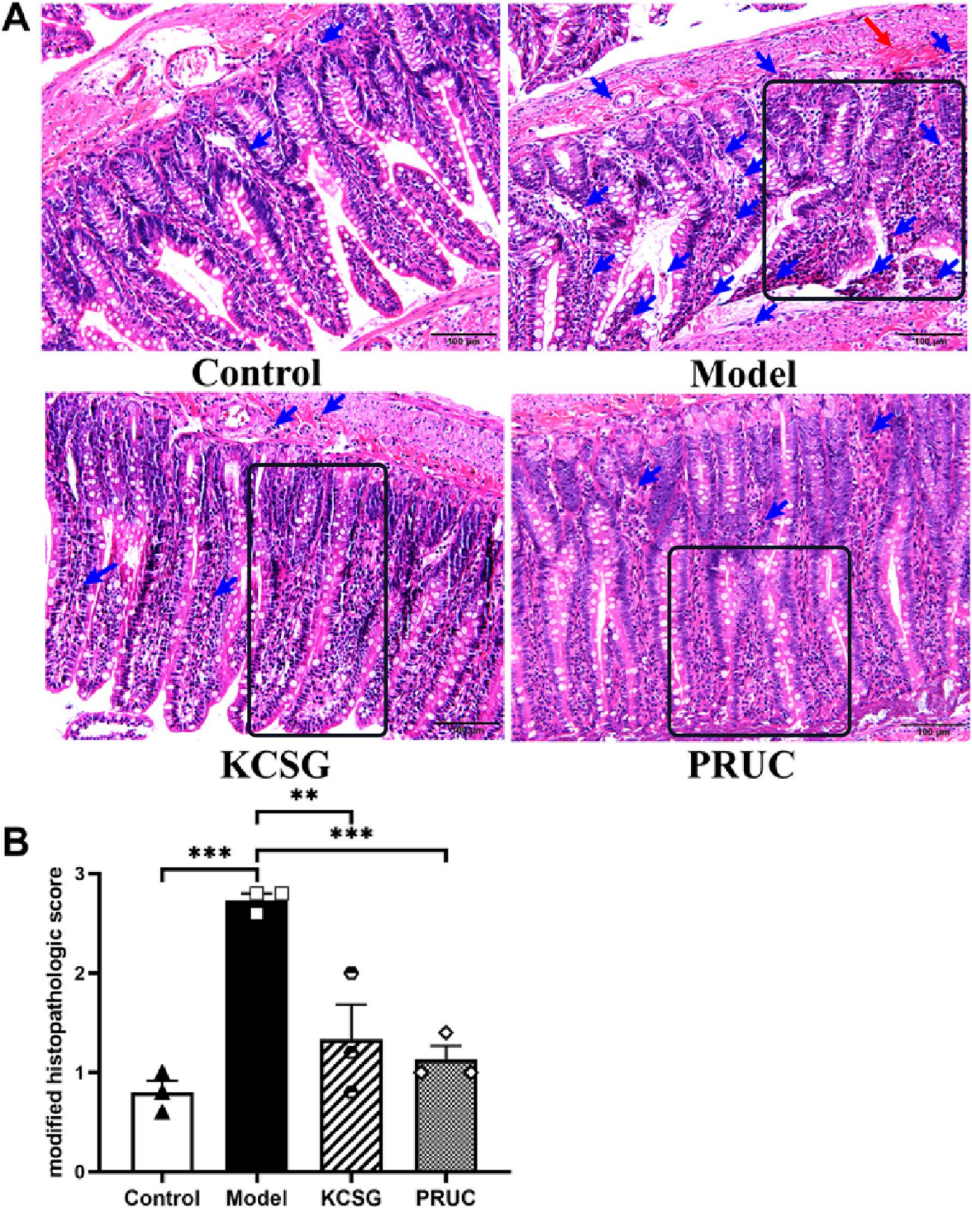
KCSG exerts protective effects on intestinal barrier in intestinal manipulation (IM)-induced POI rat. **A** Representative H&E staining of the intestinal tissues from different treatment groups at ×200 magnification (scale bar = 100 µm). Black square, red arrow and blue arrow indicate focal edema, hemorrhage or necrosis of the villi as well as the inflammatory response with infiltration of macrophages and neutrophils, respectively. **B** Histological damage score for the intestinal tissues, n = 3. Data were presented as mean ± SEM. **P < 0.01, ***P < 0.001 vs. Model group

As shown in Fig. 7A, the intestinal tissues of rats in the control group showed normal architecture with inflammatory cells in a normal level. While focal edema (black square), hemorrhage or necrosis of the villi (red arrow) as well as inflammatory reaction owing to infiltration of neutrophil and macrophage cells in the submucosa (blue arrow) could be observed in the POI group, which suggest that the intestinal endothelial barrier was damaged. After the treatment with KCSG for successive 4 days and PRUC for one day, these observations were significantly reduced (Fig. 7A) and evaluated by histological damage score (*p* < 0.01; Fig. 7B).

#### Recovery of IM-induced GI transport disorder by treatment of KCSG

**Fig. 8:**
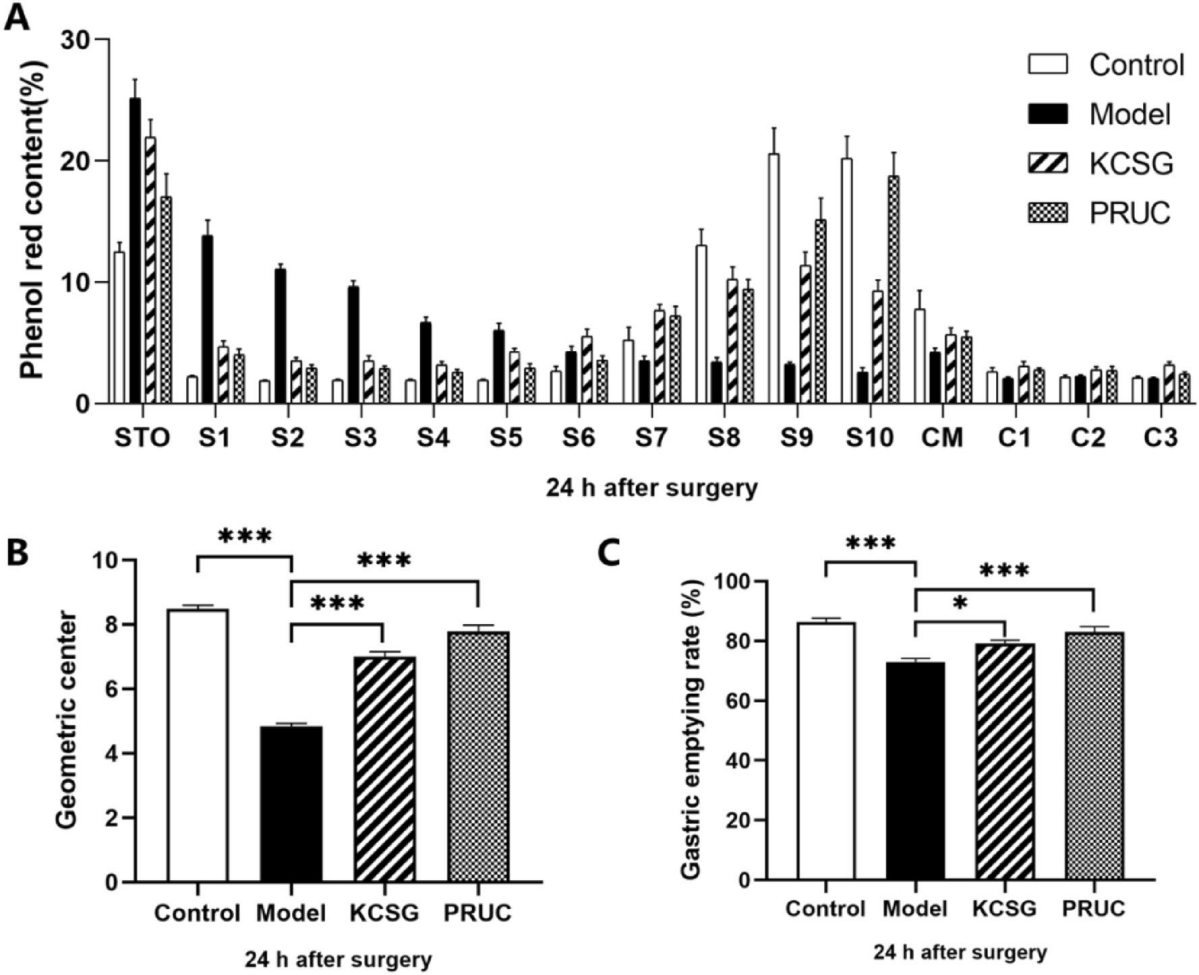
Ameliorative effect of KCSG on intestinal transit and GE in POI model rats. **A** The distribution of phenol red stayed in the stomach at 24 h. **B**, **C** The geometric center and rate of gastric emptying calculated according to the distribution of phenol red. Data were presented as mean ± SEM. *P < 0.05, **P < 0.01, ***P < 0.001 by Tukey–Kramer test, vs. Model group

In the control group, approximately 12.5% of phenol red stayed in the stomach, and 87.5% was transmitted to the intestine, peaking at S7-CM (Fig. 8A). Compared with control group, IM significantly shortened the distance of phenol red migration at 24 h after abdominal surgery. As shown in the histogram, approximately 25.2% of phenol red stayed in the stomach, whereas 74.8% was transmitted to the distal ileum, cecum and colon, peaking at S7-CM in the model group (Fig. 8A). The delayed intestinal transport caused by IM tended to recover with the treatment of KCSG and PRUC. Approximately 21.9% of phenol red stayed in the stomach, whereas 78.1% was transmitted to the distal ileum, peaking in S7-CM in the KCSG group (Fig. 8A). And approximately 17% of phenol red stayed in the stomach, whereas 83% was transmitted to the distal ileum, peaking in S10 in the PRUC group.

GC value and GE rate are also indicators for evaluating gastrointestinal transport capacity which individually represent the geometric center of the distribution of phenol red and gastric emptying ability of rats. Their values indicate the decrease and recovery of transport capacity in rat gastrointestinal, respectively. As shown in the histogram, the GC value of model group (4.8 ± 0.09) was significantly lower than that of sham-operated rats (8.48 ± 0.11) (*P* < 0.001), which was significantly recovered (*P* < 0.001) by the KCSG (7.00 ± 0.14) and PRUC (7.78 ± 0.19) treatment. Similarly, the GE rate of model group (73.01 ± 1.13%) was significantly lower than that of sham-operated rats (86.39 ± 1.25%) (P < 0.001), which was elevated in KCSG group (79.23 ± 1.00%, P < 0.05) and PRUC group (82.94 ± 1.89%, P < 0.001) (Fig. 8B and C). These results demonstrated that KCSG plays a crucial therapeutic role in POI.

#### Recovery of MPO levels in the intestinal muscle layer by treatment of KCSG

**Fig. 9:**
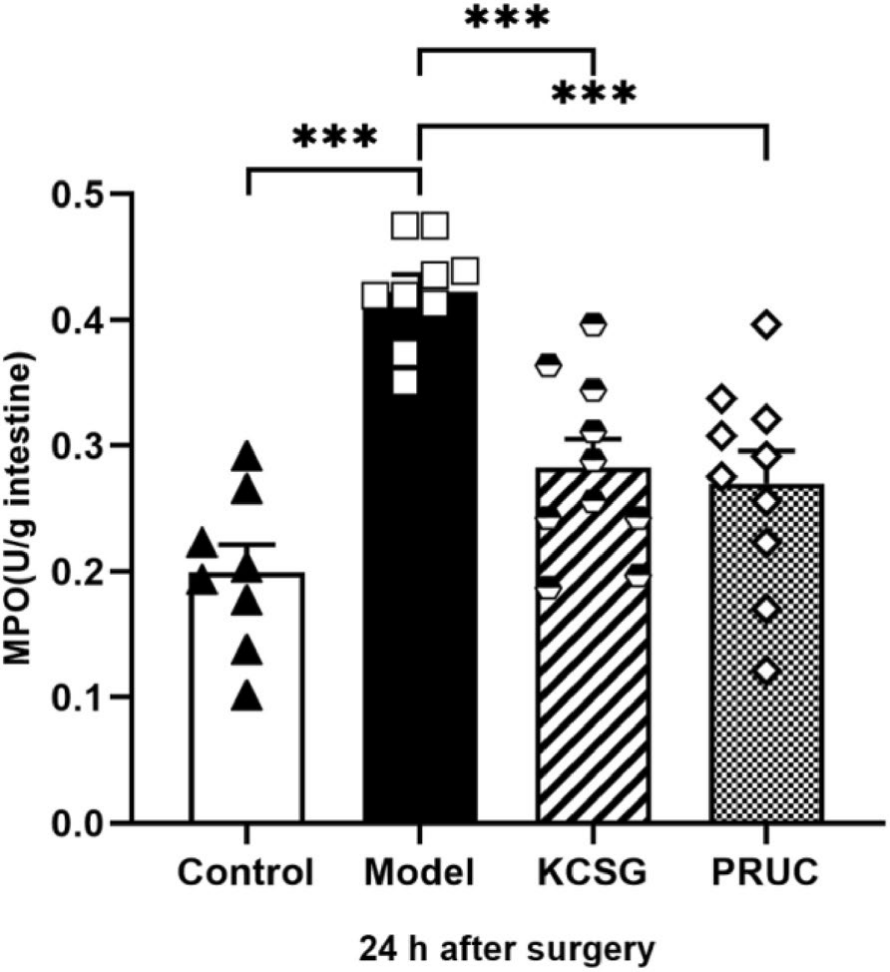
Influence of KCSG and PRUC on intestinal manipulation induced inflammation. MPO activity of the intestinal muscular layer measured 24 h after intestinal manipulation (IM) is displayed for the different groups. The MPO activity in the model group was significantly higher than that of the control group. After treatment with KCSG and PRUC, the rats' MPO activity was markedly lower than that of model rats. Data represent the means ± SEM (n = 10). ***P < 0.001 for comparison with non-treated IM rats with one-way ANOVA followed by a Bonferroni multiple comparison test

The accumulation level of MPO in the distal ileum was evaluated to validate the results of histological evaluation of neutrophils. The results suggested that the MPO level of the model rats (0.42 ± 0.01 U/g) was higher than that in the normal rats (0.20 ± 0.02 U/g) (*P* < 0.001), while KCSG group (0.28 ± 0.02 U/g) and PRUC group (0.27 ± 0.03U/g) show lower data (P < 0.001). The results clearly demonstrated that the level of MPO was significantly increased by standardized intestinal manipulation and reduced with the treatment of KCSG and PRUC (Fig. 9).

#### Western blot analysis

**Fig. 10:**
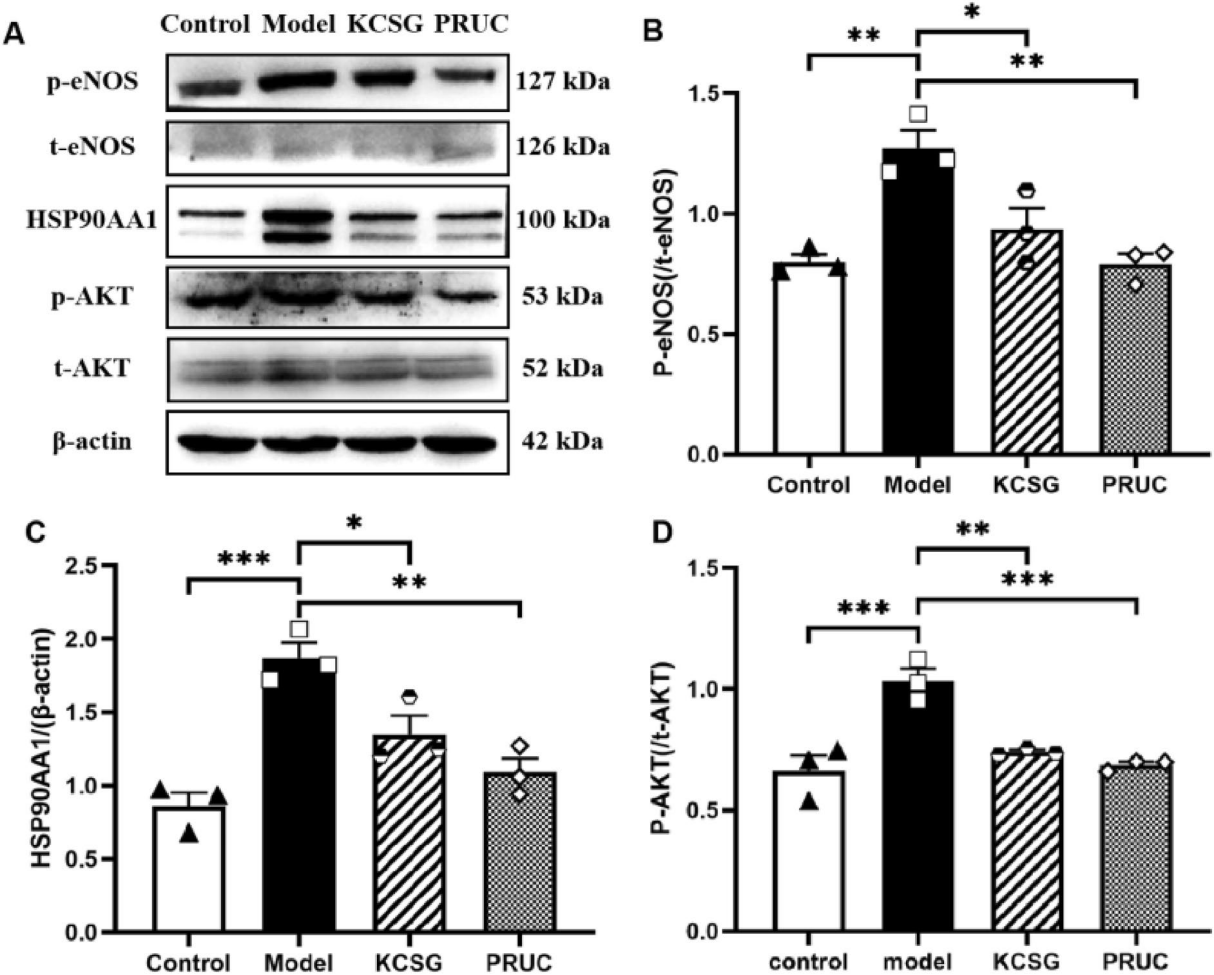
KCSG protect intestinal muscular layer of SD rats by regulated AKT/HSP90AA1/eNOS signaling pathway. **A** Western blot analysis of eNOS, AKT and HSP90AA1 in rat intestinal tissue from different groups. **B**–**D** Statistical analysis of the effects of KCSG on protein expressions levels. Data are presented as the mean ± SEM (n = 3), *P < 0.05, **P < 0.01, ***P < 0.001 vs Model

As shown in Fig. 10, the level of p-AKT/AKT and HSP90AA1 in rat distal ileum muscle layer in the model group was increased than that in the sham group, which was markedly down-regulated after the treatment of KCSG and PRUC. Conversely, the level of basal p-eNOS/eNOS was up-regulated in the POI rats and notably down-regulated by the treatment of KCSG and PRUC. Above results further evaluated and validated the screened results of network analysis.

## Discussion

As a common complication after intra-abdominal surgery, the pathological mechanisms of POI are very complicated, which have been associated with neurogenic, inflammatory and inflammatory-neuronal interactive mechanisms [41]. With the activation of inflammatory stimulation and mechanical stress caused by IM, inflammatory cytokines, chemokines, nitric oxides and prostaglandins are produced by many macrophages, which eventually lead to gastrointestinal motility disorders [42–46].

The pharmacological research in treatment of POI has gained extensively attention in recent studies. Compared to single-target chemical drugs, TCM prescriptions that composed of diversified herbs with different efficacy have the advantages of multi-component, multi-pathway, and multi-target cooperation. According to TCM theory, the etiopathogenesis of POI belongs to the domain of qi stagnation and blood stasis, syndrome of heat accumulated in large intestine, which should be treated according to the principle of regulating qi, invigorating the circulation of blood as well as relaxing the bowels. KCSG stemmed from the classic prescription DCQD is composed of 10 herbs, which have been clinically applied for a long time with a meaningful role in treating POI. Nevertheless, the comprehensive and systematic research on its material basis and overall mechanism of action has not yet been carried out.

A total of 246 components were firstly characterized by UHPLC-QTOF-MS analysis, of which 60 components was in accorded with the Lipinski’s rule of five and mainly belong to alkaloids, flavonoids, and organic acids. Subsequently, 41 core constituents were screened out by NP, which included 13 quinones, 10 flavonoids, 5 terpenoids, 4 phenols, 3 alkaloids, 2 phenylpropanoids, 1 organic acid, 1 aldehyde and 2 miscellaneous compounds, and mainly from MOC, SMR and RRR. The previous studies found that flavonoids (such as quercitrin) were suggested to have iNOS inhibition to inhibit the excessive production of NO during intestinal inflammation and mitigate the enterotoxicity [47, 48]. In addition, extensive research has shown that honokiol (**70**) could inhibit the mRNA expression of iNOS and proinflammatory cytokines. As a consequence, gastrointestinal dysmotility and intestinal inflammation were significantly recovered and inhibited in postoperative ileus, respectively [49]. Moreover, the colitis of rodent was attenuated by Salviae Miltiorrhizae with regulating gut microflora, intestinal integrity, immune conditions, cell death, free radicals, and cytokines [50]. The intestinal barrier of diabetic mice was strengthened by bioactive compounds of Salviae Miltiorrhizae such as salvianolic acid (Salvianolic acid C, **111**; Salvianolic acid F, **129**; Salvianolic acid B, **151**; Salvianolic acid A, **153**; Salvianolic acid D, **174**) through up-regulating the expression of tight junction proteins (Claudin-5, ZO-1 and Occludin) in ileum and colon [51, 52]. Also, rhein (**204**) has the potential effects to promote goblet cell proliferation within the intestinal mucosa, decrease intestinal permeability, suppress intestinal inflammation, and scavenge oxygen-free radicals to protect and maintain intestinal epithelial barrier [53, 54].

The potential targets of the effective components of KCSG in treating POI were analyzed through NP. The enrichment analysis of KEGG suggested that 41 core constituents of KCSG regulate the signal transduction of 15 pathways via 24 key targets. The key targets were closely related to inflammatory response, angiogenesis, cell proliferation and apoptosis. And VEGF signaling pathways were finally screened out to be the most qualified. In addition, the results of MD showed that relatively important targets including AKT1, eNOS and HSP90AA1 had a good matching degree with the structure of 41 key compositions.

The production of NO is crucial for the up-regulation of the inflammation in splanchnic ischemia/reperfusion and partakes in end-organ injury under such circumstances [38]. High levels of NO are even associated with cytotoxicity [55]. ENOS is a rate-limiting enzyme, which participates in NO synthesis by oxidizing the terminal guanidine nitrogen atom of l-arginine to NO [56]. The phosphorylation of eNOS at the allelic sites of S1177 and S633 was activated by AKT to regulate the endothelial cell proliferation, participate in inflammation and damage of tissues and organs and intestinal transit [56, 57]. HSP90AA1 is an inducible molecular chaperone with the function of the homodimer. The ATPase activity is regulated by HSP90AA1 which cooperates with a companion for the correct folding of specific target proteins. In this study, HSP90 in coordination with AKT activates eNOS to regulate HIF-1α and affect the expression of VEGF [56, 58]. To sum up, AKT1/HSP90AA1/eNOS may be a potential pathway for treating POI.

As for the active ingredient screened in this study with high binding activity to eNOS, the marmin (**193**) derived from AFI has kinds of activities such as anti-tumor, neuroprotective, anti-inflammatory, free radical scavenging, and anti-gastric ulcer. Some researches shows that marmin (**193**) has obvious cytoprotective activity on gastric injury induced by ethanol, which mainly maintains cellular integrity of the gastrointestinal mucosa to prevent strong stimulation by increasing the synthesis of mucosal prostaglandins [59, 60]. In addition, marmin (**193**) can inhibit the expression of eNOS by interfering with histamine receptors and inhibiting the release of histamine from mast cells [61]. RS, has the functions of digestion, detumescence, qi lowering and phlegm resolving, and sinapine thiocyanate (**45**) servses as its active ingredient. The research has shown that sinapine thiocyanate (**45**) could downregulate the expression of coagulation factors in dysfunctional vascular endothelial cells, inhibit the pre-thrombotic state caused by endothelial inflammation damage, reduce gastrointestinal pressure, and promote gastrointestinal transport [62]. Besides, magnolignan E (**198**) is the active ingredient with the high binding activity to HSP90AA1, while its function and mechanism of action still need to be explored.

**Fig. 11:**
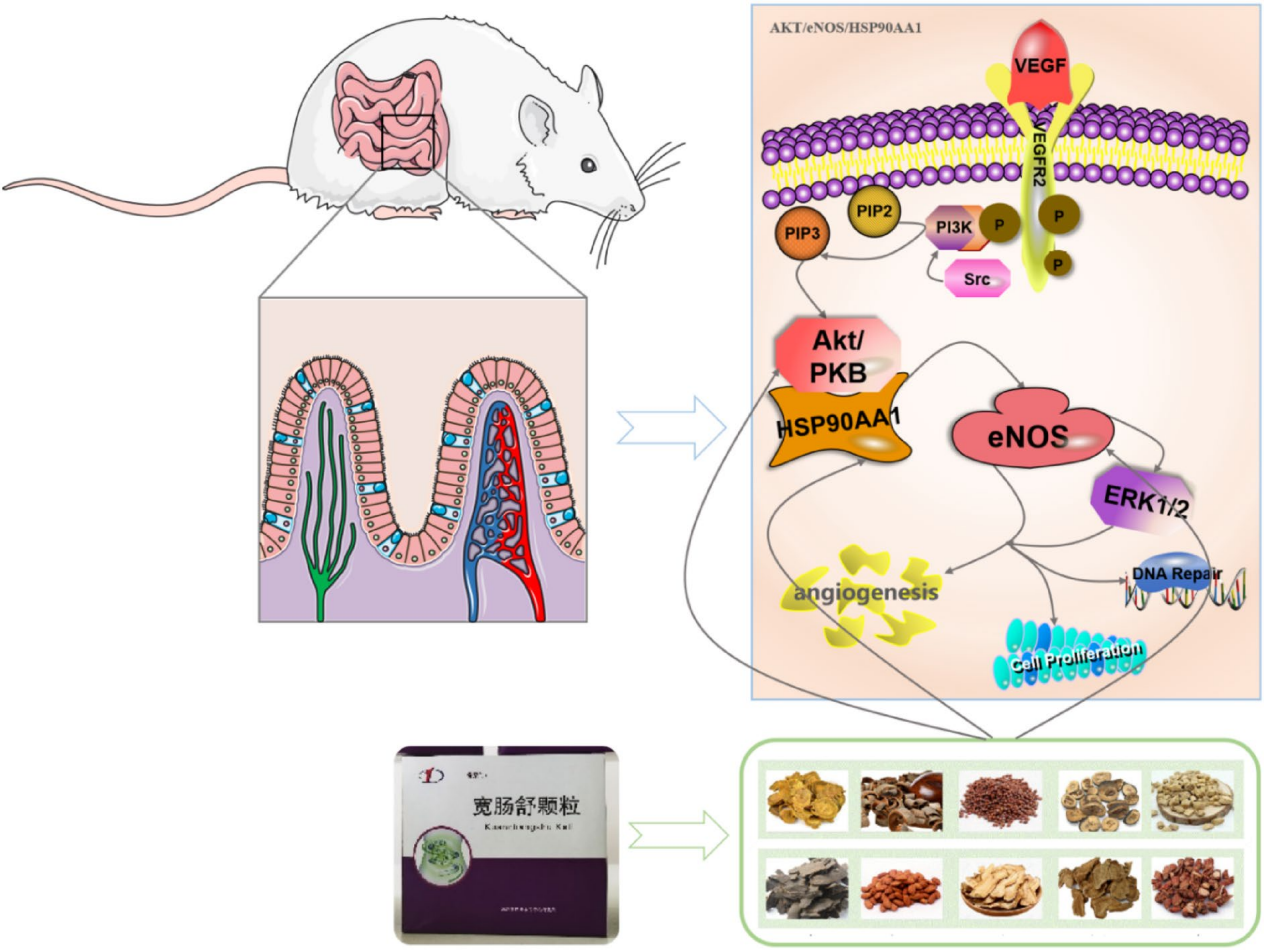
Schematic model for the molecular mechanisms associated with the protective effects of KCSD on IM-induced POI

IM-induced SD rat model were used to verify the analysis results of the network pharmacologic analysis and MD. KCSG treatment could inhibit the expression level of AKT and the phosphorylation of p-eNOS and p-HSP90AA1 and subsequently reduce the level of NO, inhibit angiogenesis, anti-inflammatory and promote intestinal transport. Experimental method combined with literature researches demonstrated that POI could be treated by KCSG through AKT/eNOS/HSP90AA1 pathway (Fig. 11).

## Conclusion

The incidence of POI remains high-level in recent years. With the wider clinical application, TCM has a good effect after long-term clinical application in treating patients of POI. In this research, UHPLC-QTOF-MS, network pharmacology, molecular docking and experimental validation in vivo was utilized to systematically expose the therapeutic mechanisms of KCSG in treating POI. It was demonstrated that AKT/HSP90AA1/eNOS signaling pathway contributed greatly to the inhibitory effect of KCSG on POI. Previously, POI was mostly treated through anti-inflammatory and promoting gastrointestinal motility. However, this study revealed that enterotoxin caused by a large amount of NO production may aggravate POI, which may provide a new idea for POI research (Additional files 2, 3, 4).

In addition, there are still many shortcomings in this study. Firstly, as the active ingredients and targets with the highest binding activity, NOS3-marmin, AKT-sinapine thiocyanate and HSP90AA1-magnolignan E have not been validated by inhibitors or targeted drugs. Some specific binding experiments such as Surface Plasma Resonance (SPR) are still needed to observe their interaction process between two pairs and detect their affinity. Secondly, the single dose selected for animal administration in this experiment was determined based on clinical application. However, three doses including low-dose, medium-dose and high-dose should be set to reflect the dose–effect relationship scientifically in pharmacological research. Thirdly, except for in vivo validations, some in vitro experiments such as small intestine smooth muscle cell experiments [9] and intestinal smooth muscle movement experiments should be used to evaluate the efficacy of drug monomers and validate relevant mechanisms. Fourthly, POI is caused by a combination of multiple mechanisms, and TCM plays its role through the complex interaction between various chemical components. Therefore, the research on upstream and downstream pathways and other efficacy and mechanisms of KCSG in treating POI still requires our attention and exploration. In summary, all these questions need to be taken into account in future research. Therefore, a comprehensive cure of POI still requires the joint efforts of researchers in various fields.

### Supplementary Information

**Additional file 1.** Characterization of the chemical constituents in KCSG by UHPLC–QTOF MS.

**Additional file 2.** The code of active ingredients of KCSG.

**Additional file 3.** The relationship between the key active ingredients of KCSG and core targets of key active ingredients.

**Additional file 4.** The relationship between pathways and the core targets with the active ingredients of KCSG.

## Data Availability

The datasets and all the relevant codes are available from the corresponding author.

## Abbreviations

ASR: Angelicae Sinensis Radix
BPC: Base peak chromatogram
AFI: Aurantii Fructus Immaturus
CM: Cecum
CON: Control
COVID-19: Coronavirus disease 2019
CR: Codonopsis Radix
AR: Aucklandiae Radix
DCQD: Da-Cheng-Qi Decoction
GC: Geometric center
GIT: Gastrointestinal transit
GE: Gastric emptying
H&E: Hematoxylin and eosin
ICCs: Interstitial cells of Caja
IM: Intestinal manipulation
KCSG: Kuanchang-Shu granule
MD: Molecular docking
MOC: Magnolia officinalis Cortex
MPO: Myeloperoxidase
XOD: Optical densities
POI: Postoperative ileus
PS: Persicae Semen
PPI: Protein–Protein Interaction
PRUC: Prucalopride
RRR: Rhei Radix et Rhizome
RS: Raphani Semen
SD: Sprague–Dawley
SMR: Salviae Miltiorrhizae Radix et Rhizoma
SR: Scrophulariae Radix*.*
SPR: Surface Plasma Resonance
STO: Stomach
TCM: Traditional Chinese medicine
TCMSP: Traditional Chinese Medicine Systems Pharmacy Database and Analysis Platform
TRPA1: Transient receptor potential A1
UHPLC-QTOF-MS: Ultra-performance liquid chromatography coupled with quadrupole time-of-flight mass spectrometry
VEGF: Vascular endothelial growth factor
VIP: Vasoactive intestinal peptide
WB: Western blotting
