# Supplementary material for: Exploring the chemical components of Kuanchang-Shu granule and its protective effects of postoperative ileus in rats by regulating AKT/HSP90AA1/eNOS pathway
Source: Chin Med. 2024 Feb 21;19:29. doi: 10.1186/s13020-024-00892-3 (PMC10880223; doi:10.1186/s13020-024-00892-3)
Supplement: Supplementary file 1 — Additional file 1. Characterization of the chemical constituents in KCSG by UHPLC–QTOF MS. [file 13020_2024_892_MOESM1_ESM.docx]

**Table S1. Characterization of the chemical constituents in KCSG by UHPLC–QTOF MS**

Peak t_R_ Identification Formula Negative mode Positive mode Source *^a^*

No (min) Quasi-molecular Observed Calculated ppm Fragement ions Quasi-molecular Observed Calculated ppm Fragement ions

ion mass (Da) mass (Da) ion mass (Da) mass (Da)

1 0.77 2'-Deoxyadenosine C_10_H_13_N_5_O_3_ [M-H]^-^ 250.0929 250.0946 -6.8 — — — — — — ASR

2 0.77 Arginine C_6_H_14_N_4_O_2_ [M-H]^-^ 173.1040 173.1044 -2.3 156.3940[M-H-NH_3_]^-^ [M+H]+ 175.1177 175.1190 -7.4 158.0870[M+H-NH_3_]^+^ CR/SR/ASR

131.0850[M-H-C_3_H_6_]^-^ 130.0918[M+H-COOH]^+^

70.0610[M+H-NaH_3_-C_3_H_6_NO_2_]^+^

60.0515[M+H-C_5_H_10_NO_2_]^+^

3 0.79 Verbascose C_30_H_52_O_26_ [M-H]^-^ 827.2685 827.2674 1.3 — [M+H]^+^ 829.2890 829.2820 8.4 — CR

[M+Na]^+^ 851.2603 851.2639 -4.2

4 0.81 β-D-Glucopyranose C_6_H_12_O_6_ [M-H]^-^ 179.0558 179.0561 -1.7 103.3352[M-H-C_2_H_4_O_3_]^-^ — — — — — RS

[M+CH_3_COO]^-^ 239.0764 239.0772 -3.4

5 0.81 Aspartic acid C_4_H_7_NO_4_ [M-H]^-^ 132.0314 132.0302 9.1 115.0037[M-H-NH_3_]^-^ [M+H]^+^ 134.0440 134.0448 -6.0 116.0383[M+H-H_2_O]^+^ SR

89.0244[M-CO_2_]^-^ 88.0303[M+H-HCOOH]^+^

71.0140[M-H-CO_2_-NH_3_]^-^ 74.0144[M+H-NH_3_-CO-CH_3_]^+^

6 0.81 Lactic acid C_3_H_6_O_3_ [M-H]^-^ 89.0242 89.0244 -2.2 — — — — — — ASR

7 0.81 Serine C_3_H_7_NO_3_ [M-H]^-^ 104.0350 104.0353 -2.9 — — — — — — RS/ASR

8 0.81 Trans-4-Hydroxy-L-proline C_5_H_9_NO_3_ [M+CH_3_COO]^-^ 190.0729 190.0721 4.2 — — — — — — ASR

9 0.82 Tanshindiol C C_18_H_16_O_5_ [M-H]^-^ 311.0955 311.0925 9.6 — — — — — — SMR

[M+HCOO]^-^ 357.0988 357.0980 2.2

10 0.82 Hypoxanthine C_5_H_4_N_4_O [M-H]^-^ 135.0311 135.0312 -0.7 — — — — — — ASR

11 0.82 Trimethoxyflavone C_18_H_16_O_5_ [M-H]^-^ 311.0935 311.0925 3.2 — — — — — — AFI

[M+HCOO]^-^ 357.0987 357.0980 2.0

12 0.83 Delta-D-Mannonolactone C_6_H_10_O_6_ [M-H]^-^ 177.0401 177.0405 -2.3 — — — — — — ASR

[M+HCOO]^-^ 223.0456 223.0459 -1.4

13 0.91 Sinapine C_16_H_24_NO_5_ [M-H]^-^ 309.1578 309.1582 -1.3 — — — — — — RS

[M+HCOO]^-^ 355.1634 355.1637 -0.8

14 1.02 Rhamnazin 3-Rutinoside C_29_H_34_O_16_ [M-H]^-^ 637.1806 637.1774 5.0 — — — — — — MOC

15 1.04 Obovatal C_18_H_16_O_4_ [M+Cl]^-^ 331.0724 331.0743 -5.7 178.0250[M-H-CH_9_]^-^ — — — — — MOC

[M+HCOO]^-^ 341.1045 341.1031 4.1 150.0270[M-H-C_9_H_9_-CO]^-^

16 1.07 Acetic acid C_2_H_4_O_2_ [M-H]^-^ 59.0138 59.0139 -1.7 — — — — — — ASR

[M+CH_3_COO]^-^ 119.0347 119.0350 -2.5

17 1.10 Lysine C_6_H_14_N_2_O_2_ — — — — — [M+Na]^+^ 169.0947 169.0947 0.0 130.0856[M+H-NH_3_]^+^ SR/ASR

84.0704[M+H-NH_3_-HCOOH]^+^

18 1.20 Citric acid C_6_H_8_O_7_ [M-H]^-^ 191.0203 191.0197 3.1 173.0051[M-H-H_2_O]^-^ — — — — — MOC/AFI/ASR

147.0253[M-H-CO_2_]^-^

129.0151[M-H-CO_2_-H_2_O]^-^

111.0057[M-H-CO_2_-H_2_O-H_2_O]^-^

87.0061[M-H-CO_2_-H_2_O-CO-CH_2_]^-^

85.0265[M-H-CO_2_-H_2_O-CO_2_]^-^

19 1.22 Aloesone C_13_H_12_O_4_ [M-H]^-^ 231.0679 231.0663 6.9 — — — — — — RRR

[M+HCOO]^-^ 277.0721 277.0718 1.1

20 1.22 Pyroglutamic acid C_5_H_7_NO_3_ [M+CH_3_COO]^-^ 188.0561 188.0564 -1.6 — — — — — — SR

21 1.22 Torachrysone C_14_H_14_O_4_ [M+HCOO]^-^ 291.0876 291.0874 0.7 — [M+H]^+^ 247.0957 247.0965 -3.2 — RRR

[M+Na]^+^ 269.0799 269.0784 5.6

22 1.30 Quinic acid C_7_H_12_O_6_ [M+HCOO]^-^ 237.0623 237.0616 3.0 127.0343[M-H-2H_2_O-CO]^-^ — — — — — AFI

[M+CH_3_COO]^-^ 251.0766 251.0772 -2.4 109.0251[M-H-3H_2_O-CO]^-^

93.0337[M-H-3H_2_O-CO_2_]^-^

23 1.34 Uridine C_9_H_12_N_2_O_6_ [M-H]^-^ 243.0607 243.0623 -6.6 — — — — — — ASR

[M+Cl]^-^ 279.0389 279.0389 0.0

24 1.49 Daidzein 7-O-beta-D-

Glucoside 4-O-methylate C_22_H_22_O_9_ [M-H]^-^ 429.1192 429.1191 0.2 475.1320[M+HCOO]^-^ — — — — — ASR

[M+CH_3_COO]^-^ 489.1420 489.1402 3.7 267.0725[M-Glu]^-^

25 1.49 Formononetin 7-O-glucoside C_22_H_22_O_9_ [M-H]^-^ 429.1192 429.1191 0.2 — — — — — — CR

[M+CH_3_COO]^-^ 489.1420 489.1402 3.7

26 1.56 L-Ornithine C_5_H_12_N_2_O_2_ — — — — — [M+H]^+^ 133.0966 133.0972 -4.5 — ASR

27 1.57 Isoleucine C_6_H_13_NO_2_ [M-H]^-^ 130.0873 130.0874 -0.8 — [M+H]^+^ 132.1012 132.1019 -5.3 165.0373[M+H+CH_3_+H_2_O]^+^ RS/ASR

28 1.79 Tanshinol B C_18_H_16_O_4_ [M+HCOO]^-^ 341.1058 341.1031 7.9 280.0711[M-H-CH_3_]^-^ — — — — — SMR

277.0780[M-H-H_2_O]^-^

262.0380[M-H-CH_3_-H_2_O]^-^

29 1.80 3′,4′,6,7‐tetramethoxyflavone C_19_H_18_O_6_ [M-H]^-^ 341.1066 341.1094 -8.2 179.0483[M-H-C_9_H_8_O-2CH_3_]^-^ — — — — — AFI

[M+Cl]^-^ 377.0830 377.0797 8.8

[M+HCOO]^-^ 387.1117 387.1085 8.3

30 1.80 5-Hydroxy-3,3',4',6,7

**Table S1. Characterization of the chemical constituents in KCSG by UHPLC–QTOF MS**

Peak t_R_ Identification Formula Negative mode Positive mode Source *^a^*

No (min) Quasi-molecular Observed Calculated ppm Fragement ions Quasi-molecular Observed Calculated ppm Fragement ions

ion mass (Da) mass (Da) ion mass (Da) mass (Da)

-pentamethoxyflavone C_20_H_20_O_8_ [M-H]^-^ 387.1116 387.1085 8.0 341.1111[M-H-H_2_O-CO]^-^ [M+H]^+^ 389.1208 389.1231 -5.9 374.0971[M+H-CH_3_]^+^ AFI

[M+Na]^+^ 441.1058 441.1050 1.8 289.0630[M+H-3CH_3-_CO-H_2_O]^+^

183.0472[M+H-C_10_H_10_-O_2_-CO_2_]^+^

163.0524[M+H-C_10_H_10_O_2_-2CH_2_-2H_2_O]^+^

31 1.80 Methylophiopogonanone A C_19_H_18_O_6_ [M+Cl]^-^ 377.0827 377.0797 8.0 — — — — — — SR

[M+HCOO]^-^ 387.1116 387.1085 8.0

32 1.85 5-HMF C_6_H_6_O_3_ [M-H]^-^ 125.0242 125.0244 0.0 109.0532[M-H-H_2_O]^-^ — — — — — SR

81.0304[M-H-H_2_O-CO]^-^

53.0373[M-H-H_2_O-CO-CO]^-^

33 1.85 Gallic acid C_7_H_6_O_5_ [M-H]^-^ 169.0149 169.0142 4.1 125.0248[M-H-CO_2_]^-^ [M+H]^+^ 171.0291 171.0288 1.8 153.0185[M+H-H_2_O]^+^ SMR/MOC/RRR

97.0303[M-H-CO_2_-CO]^-^

107.0142[M-H-CO_2_-H_2_O]^-^

81.0346[M-H-CO_2_-H_2_O-C_2_H_2_]^-^

34 2.20 Galactinol or Maltose C_12_H_22_O_11_ [M-H]^-^ 341.1091 341.1089 0.6 — — — — — — MOC/RS

[M+HCOO]^-^ 387.1138 387.1144 -1.6

35 2.21 Fumaric acid C_4_H_4_O_4_ [M-H]^-^ 115.0037 115.0037 0.0 — — — — — — SR

36 2.40 Butanoic acid C_4_H_8_O_2_ [M+CH_3_COO]^-^ 147.0655 147.0663 -5.4 — [M+Na]^+^ 111.0415 111.0417 -1.8 — ASR

37 2.40 Ethyl propanoate C_5_H_10_O_2_ [M+HCOO]^-^ 147.0655 147.0663 -5.4 — — — — — — MOC

38 2.42 Tyrosol C_8_H_10_O_2_ [M+Cl]^-^ 173.0411 173.0394 9.8 — — — — — — MOC

39 2.69 Furfural C_5_H_4_O_2_ [M+HCOO]^-^ 141.0190 141.0193 -2.1 — — — — — — ASR

40 3.57 3,5-dihydroxybenzoic acid C_7_H_6_O_4_ [M-H]^-^ 153.0190 153.0193 -2.0 109.0292[M-H-CO_2_]^-^ — — — — — SMR

[M+CH_3_COO]^-^ 215.0384 215.0405 -9.8

41 3.57 5-Methylfurfural C_6_H_6_O_2_ [M-H]^-^ 109.0294 109.0295 -0.9 — — — — — — ASR

42 3.61 Caftaric acid C_13_H_12_O_9_ [M+CH_3_COO]^-^ 371.0610 371.0620 -2.7 — — — — — — SMR

43 3.62 Methyl Linolenate C_19_H_32_O_2_ [M+Cl]^-^ 329.2251 329.2253 -0.6 — [M+H]^+^ 293.2473 293.2475 -0.7 — RS

[M+CH_3_COO]^-^ 351.2516 351.2541 -7.1

44 3.84 Protocatechuic acid C_7_H_6_O_4_ [M-H]^-^ 153.0191 153.0193 -1.3 109.0273[M-H-CO_2_]^-^ — — — — — ASR

81.0351[M-H-CO_2_-CO]^-^

53.0391[M-H-CO_2_-CO-CO]^-^

45 4.16 Sinapine thiocyanate C_16_H_24_NO_5_CNS [M+Cl]^-^ 403.1103 403.1100 0.7 — — — — — — RS

[M+CH_3_COO]^-^ 428.1626 428.1623 0.7

46 4.29 Harpagide C_15_H_24_O_10_ [M-H]^-^ 363.1304 363.1297 1.9 201.0753[M-H-Glc]^-^ — — — — — SR

[M+HCOO]^-^ 409.1366 409.1351 3.7 183.0653[M-H-Glc-H_2_O]^-^

165.0533[M-H-Glc-H_2_O-H_2_O]^-^

139.0382[M-H-Glc-H_2_O-H_2_O-C_2_H_2_]^-^

47 4.71 L-Tryptophan C_11_H_12_N_2_O_2_ [M-H]^-^ 203.0827 203.0826 0.5 — — — — — — ASR/PS/MOC

[M+CH_3_COO]^-^ 263.1069 263.1057 4.6

48 5.08 Inosine C_10_H_12_N_4_O_5_ [M+HCOO]^-^ 313.0806 313.0790 5.1 — — — — — — ASR

49 5.20 Isomucronulatol 7-O-glucoside C_23_H_28_O_10_ [M-H]^-^ 463.1639 463.1610 6.3 303.2228[M-H-CO-3CO_2_]^-^ — — — — — CR

167.0339[M-H-C_14_H_6_O_7_]^-^

123.0136[M-H-C_16_H_20_O_8_]^-^

50 5.44 P-hydroxybenzoic acid-

O-galloyl-glucoside C_7_H_6_O_3_ [M-H]^-^ 137.0242 137.0244 -1.5 109.0291[M-H-CO]^-^ — — — — — RRR

[M+CH_3_COO]^-^ 197.0458 197.0455 1.5 93.0330[M-H-CO_2_]^-^

51 5.45 9,10-Dimethoxypterocarpan-3-

O-Beta-D-Glucoside C_23_H_26_O_10_ [M-H]^-^ 461.1440 461.1453 -2.8 — — — — — — ASR

52 5.53 (E)-THSG C_20_H_22_O_9_ [M+HCOO]^-^ 451.1248 451.1246 0.4 — [M+Na]^+^ 429.1169 429.1156 3.0 — CR

53 5.76 P-Hydroxybenzoic acid C_7_H_6_O_3_ [M-H]^-^ 137.0243 137.0244 -0.7 81.0335[M-H-CO-CO]^-^ — — — — — RRR

[M+CH_3_COO]^-^ 197.0440 197.0455 -7.6 66.0348[M-H-CO-CO-CH_3_]^-^

54 5.89 Ketoleucine C_6_H_10_O_3_ [M+HCOO]^-^ 175.0611 175.0612 -0.6 — — — — — — RRR

55 6.33 Catechin or (-)-Epicatechin C_15_H_14_O_6_ [M-H]^-^ 289.0730 289.0718 4.2 165.0103[M-H-C_7_H_9_O_2_]^-^ — — — — — SMR/MOC/RRR

137.0240[M-H-C_8_H_8_O_3_]^-^

245.0792[M-H-CO_2_]^-^

56 6.33 Methyl anthranilate C_8_H_9_NO_2_ [M-H]^-^ 150.0549 150.0561 -8.0 — [M+H]^+^ 152.0715 152.0706 5.9 — MOC

[M+HCOO]^-^ 196.0596 196.0615 -9.7

[M+CH_3_COO]^-^ 210.0775 210.0772 1.4

57 6.33 Piceatannol C_14_H_12_O_4_ [M+HCOO]^-^ 289.0730 289.0718 4.2 245.0835[M-H+2H]^-^ — — — — — RRR

259.0461[M-H+O]^-^

257.0062[M-H+CH_2_]^-^

**Table S1. Characterization of the chemical constituents in KCSG by UHPLC–QTOF MS**

Peak t_R_ Identification Formula Negative mode Positive mode Source *^a^*

No (min) Quasi-molecular Observed Calculated ppm Fragement ions Quasi-molecular Observed Calculated ppm Fragement ions

ion mass (Da) mass (Da) ion mass (Da) mass (Da)

58 6.62 3-Caffeoylquinic acid C_16_H_18_O_9_ [M-H]^-^ 353.0886 353.0878 2.3 191.0558[M-H-C_9_H_6_O_3_]^-^ — — — — — CR/MOC

135.0452[M-H-C_9_H_6_O_3_-CO_2_]^-^

59 6.69 Benzyl Gentiobioside C_19_H_28_O_11_ [M-H]^-^ 431.1557 431.1559 -0.4 — [M+Na]^+^ 455.1505 455.1524 -4.2 — PS

[M+HCOO]^-^ 477.1622 477.1614 1.7

60 6.92 N-Methylcoclaurine C_18_H_21_NO_3_ [M+CH_3_COO]^-^ 358.1654 358.1660 -1.7 — — — — — — MOC

61 6.93 Pipradrol C_18_H_21_NO [M+HCOO]^-^ 312.1609 312.1605 1.3 — — — — — — RRR

62 6.95 Vanillic acid C_8_H_8_O_4_ [M-H]^-^ 167.0351 167.0350 0.6 123.0451[M-H-CO_2_]^-^ — — — — — SMR/ASR

63 6.97 Malic acid C_4_H_6_O_5_ [M-H]^-^ 133.0141 133.0142 -0.8 — — — — — — ASR

64 7.04 Chlorogenic acid C_16_H_18_O_9_ [M-H]^-^ 353.0855 353.0878 -6.5 191.0557[M-H-C_9_H_6_O_3_]^-^ — — — — — SR/ASR

[M+CH_3_COO]^-^ 414.1197 414.1168 7.0 179.0323[M-H-C_7_H_10_O_5_]^-^

173.0077[M-H-C_9_H_6_O_3_-H_2_O]^-^

161.0379[M-H-C_7_H_12_O_6_]^-^

135.0492[M-H-C_7_H_10_O_5_-CO_2_]^-^

109.0393[M-H-C_7_H_10_O_5_-CO_2_-C_2_H_2_]^-^

65 7.08 Decaffeoylacteoside C_20_H_30_O_12_ [M-H]^-^ 461.1664 461.1664 0.0 — — — — — — SR

66 7.17 Aucubin C_15_H_2_O_9_ [M-H]^-^ 345.1157 345.1191 -9.9 391.5233[M+HCOO]^-^ [M+Na]^+^ 369.1170 369.1156 3.8 — SR

[M+HCOO]^-^ 391.1248 391.1246 0.5 165.0550[M-H-Glu-H_2_O]^-^

135.0611[M-H-Glu-H_2_O-HCHO]^-^

67 7.37 Amygdalin C_20_H_27_NO_11_ [M-H]^-^ 456.1523 456.1511 2.6 — — — — — — PS

[M+HCOO]^-^ 502.1586 502.1566 4.0

68 7.96 Sphingosine C_18_H_37_NO_2_ — — — — — [M+H]^+^ 300.2898 300.2897 0.3 — RRR

69 8.00 Isobutanal C_4_H_8_O [M+CH_3_COO]^-^ 131.0712 131.0714 -1.5 — — — — — — ASR

70 8.10 Honokiol C_18_H_18_O_2_ [M-H]^-^ 265.1247 265.1234 4.9 224.0843[M-H-C_3_H_5_]^-^ [M+H]^+^ 267.1386 267.1380 2.3 — MOC

[M+Cl]^-^ 301.1001 301.1001 0.0 223.0767[M-H-C_3_H_6_]^-^

[M+CH_3_COO]^-^ 325.1528 325.1524 1.2 197.0613[M-H-C_3_H_4_-CO]^-^

196.0535[M-H-C_3_H_5_-CO]^-^

71 8.13 N-Acetylnorepinephrine C_10_H_13_NO_4_ [M-H]^-^ 210.0775 210.0772 1.4 — [M+H]^+^ 196.0932 196.0944 -6.1 — AFI

[M+Na]^+^ 218.0775 218.0788 -6.0

72 8.13 Phenylalanine C_9_H_11_NO_2_ [M+HCOO]^-^ 210.0775 210.0772 1.4 165.0779[M-H] — — — — — SMR/SR/ASR

147.0034[M-H-NH_3_]^-^

72.0143[M-H-NH_3_-C_6_H_5_]^-^

73 8.20 Mandelamide C_8_H_9_NO_2_ [M-H]^-^ 150.0557 150.0561 -2.7 — — — — — — PS

[M+CH_3_COO]^-^ 210.0766 210.0772 -2.9

74 8.61 Luteolin-3',7-di-O-glucoside C_27_H_30_O_16_ [M-H]^-^ 609.1475 609.1461 2.3 301.0324[M-H-Rha-Glc]^-^ — — — — — AFI

75 8.61 Rutin or Quercetin-O-rutinoside C_27_H_30_O_16_ [M-H]^-^ 609.1475 609.1461 2.3 300.0742[M-H-C_12_H_21_O_9_]^-^ — — — — — MOC/RRR

271.0408[M-H-C_12_H_21_O_9_-CO]^-^

255.0533[M-H-C_12_H_21_O_9_-CO_2_]^-^

243.0789[M-H-C_12_H_21_O_9_-CO-CO]^-^

151.0426[M-H-C_12_H_21_O_9_-CO-C_7_H_5_O_2_]^-^

109.0287[M-H-C_21_H_24_O_14_]^-^

107.0386[M-H-C_12_H_21_O_9_-CO-C_7_H_5_O_2_-CO_2_]^-^

76 8.78 Indole C_8_H_7_N [M-H]^-^ 116.0515 116.0506 7.8 — — — — — — MOC

77 9.03 (2S)-(β-D-Glucopyranosyloxy)

(phenyl)acetonitrile C_14_H_17_NO_6_ [M-H]^-^ 294.0986 294.0983 1.0 161.0463[M-C_8_H_8_NO]^-^ — — — — — PS

[M+HCOO]^-^ 340.1052 340.1038 4.1

78 9.04 Echinacoside C_35_H_46_O_20_ [M-H]^-^ 785.2535 785.2510 3.2 623.2196[M-H-C_9_H_6_O_3_]^-^ [M+Na]^+^ 809.2454 809.2475 -2.6 — MOC

477.1604[M-H-C_9_H_6_O_3_-C_6_H_10_O_4_]^-^

161.0251[M-H-C_26_H_40_O_17_]^-^

79 9.30 Apigenin-6,8- di-C-glucoside C_27_H_30_O_15_ [M-H]^-^ 593.1533 593.1512 3.5 473.1097[M-H-C_4_H_8_O_4_]^-^ — — — — — AFI

[M+CH_3_COO]^-^ 653.1735 653.1723 1.8 297.0813[M-H-Rha-C_8_H_6_O_2_]

80 9.44 Limetin C_11_H_10_O_4_ [M+HCOO]^-^ 251.0562 251.0561 0.4 — [M+H]^+^ 207.0632 207.0652 -9.7 — AFI

81 9.57 P-coumaric acid C_9_H_8_O_3_ [M-H]^-^ 163.0402 163.0401 0.6 119.0492[M-H-CO_2_]^-^ — — — — — SMR/SR

[M+HCOO]^-^ 209.0448 209.0455 -3.4 93.0360[M-H-CO_2_-C_2_H_2_]^-^

[M+CH_3_COO]^-^ 223.0608 223.0612 -1.8

82 9.60 4-O-beta-Glucopyranosyl-

cis-couMaric acid C_15_H_18_O_8_ [M+HCOO]^-^ 371.0982 371.0984 -0.5 163.0392[M-H-C_6_H_10_O_5_]^-^ — — — — — RRR

[M+CH_3_COO]^-^ 385.1140 385.1140 0.0 119.0490[M-H-C_6_H_10_O_5_-CO_2_]^-^

83 9.86 Kaempferol-3-O-rutinoside C_27_H_30_O_15_ [M-H]^-^ 593.1471 593.1512 -6.9 473.1092[M-H-C_4_H_8_O_4_]^-^ — — — — — SMR/MOC

297.0767[M-H-Rha-C_8_H_6_O_2_]^-^

84 10.32 Cytidine C_9_H_13_N_3_O_5_ [M+HCOO]^-^ 288.0856 288.0837 6.6 — — — — — — ASR

85 10.40 Keioside C_28_H_32_O_16_ [M-H]^-^ 623.1657 623.1618 6.3 — [M+H]^+^ 625.1737 625.1763 -4.2 — MOC

**Table S1. Characterization of the chemical constituents in KCSG by UHPLC–QTOF MS**

Peak t_R_ Identification Formula Negative mode Positive mode Source *^a^*

No (min) Quasi-molecular Observed Calculated ppm Fragement ions Quasi-molecular Observed Calculated ppm Fragement ions

ion mass (Da) mass (Da) ion mass (Da) mass (Da)

86 10.41 Hesperitin or Imperatorin C_16_H_14_O_4_ [M-H]^-^ 269.0827 269.0819 3.0 124.0197[M-H-C_9_H_9_-CO]^-^ [M+H]^+^ 271.0834 271.0863 -9.6 — AFI/ASR

87 10.58 Acteoside C_29_H_36_O_15_ [M-H]^-^ 623.2002 623.1981 3.4 461.1711[M-H-C_9_H_6_O_3_]^-^ [M+Na]^+^ 647.1928 647.1946 -2.8 — MOC/SR

161.0251[M-H-C_20_H_30_O_12_]^-^

133.0292[M-H-C_21_H_30_O_13_]^-^

88 10.65 Umbelliferone C_9_H_6_O_3_ [M+CH_3_COO]^-^ 221.0456 221.0455 0.5 133.0008[M-H-CO]^-^ — — — — — ASR

117.0596[M-H-CO_2_]^-^

105.0441[M-H-2CO]^-^

89 11.00 Obovaaldehyde C_16_H_14_O_4_ [M-H]^-^ 269.0799 269.0819 -7.4 152.0426[M-H-C_9_H_9_]^-^ — — — — — MOC

124.0337[M-H-C_9_H_9_-CO]^-^

267.2745[M-H-CO]^-^

90 11.21 Ferulic acid C_10_H_10_O_4_ [M-H]^-^ 193.0505 193.0506 -0.5 178.0263[M-H-CH_3_]^-^ [M+H]^+^ 195.0640 195.0652 -6.2 177.0516[M+H-H_2_O]^+^ SMR/SR/ASR

[M+CH_3_COO]^-^ 253.0741 253.0718 9.1 177.0204[M-H-O]^-^ [M+Na]^+^ 217.0466 217.0471 -2.3 149.0564[M+H-H_2_O-CO] ^+^

149.0600[M-H-CO_2_]^-^

145.0146[M-H-CH_4_O_2_]^-^

134.0370[M-H-C_2_H_3_O_2_]^-^

89.0401[M-H-C_6_O_2_]^-^

91 11.69 3-Ethenyl-alpha-hydroxy C_10_H_10_O_3_ [M+HCOO]^-^ 223.0608 223.0612 -1.8 — — — — — — ASR

-benzeneacetic acid [M+CH_3_COO]^-^ 237.0766 237.0768 -0.8

92 11.80 3, 4-Dihydroxybenzoic acid-O-glu C_10_H_10_O_4_ [M-H]^-^ 193.0497 193.0506 -4.7 178.0270[M-H-CH_3_]^-^ — — — — — MOC

[M+CH_3_COO]^-^ 253.0741 253.0718 9.1 149.0631[M-H-CO_2_]^-^

134.0438[M-H-CH_3_-CO_2_]^-^

93 11.87 Isorhapontin C_21_H_24_O_9_ [M+HCOO]^-^ 465.1379 465.1402 -4.9 — — — — — — RRR

94 12.15 1-O-caffeoylglucose C_15_H_18_O_9_ [M-H]^-^ 341.0888 341.0878 2.9 — — — — — — SMR

95 12.19 17-hydroxypregnenolone sulfate C_21_H_32_O_6_S [M+CH_3_COO]^-^ 471.2087 471.2058 6.2 329.0838[M-H-H_2_SO_3_]^-^ — — — — — ASR

96 12.31 Hydroxycinnamic acid C_9_H_8_O_3_ [M-H]^-^ 163.0396 163.0401 -3.1 119.0454[M-H-CO_2_]^-^ — — — — — MOC

[M+Cl]^-^ 199.0164 199.0167 -1.5

97 12.85 Pipecolic acid C_6_H_11_NO_2_ — — — — — [M+Na]^+^ 152.0675 152.0682 -4.6 — MOC/AFI

98 12.90 Tanshinone I C_18_H_12_O_3_ — — — — — [M+H]^+^ 277.0814 277.0835 -7.6 262.0625[M+H-CH_3_]^+^ SMR

[M+Na]^+^ 299.0675 299.0679 -1.3 249.0905[M+H-CO]^+^

231.0809[M+H-CO-H_2_O]^+^

221.0967[M+H-CO-CO]^+^

178.0772[M+H-CO-CO-CO-CH_3_]^+^

99 12.90 Scopoletin C_10_H_8_O_4_ [M+HCOO]^-^ 237.0414 237.0405 3.8 — — — — — — AFI/ASR

[M+CH_3_COO]^-^ 251.0562 251.0561 0.4

100 13.09 Lipedoside A C_29_H_36_O_14_ [M+Cl]^-^ 643.1804 643.1799 0.8 — — — — — — MOC

[M+CH_3_COO]^-^ 667.2259 667.2244 2.3

101 13.13 (E/Z)-Polydatin C_20_H_22_O_8_ [M-H]^-^ 389.1251 389.1242 2.3 — — — — — — RRR

[M+HCOO]^-^ 435.1305

102 13.15 6'-O-feruloylsucrose C_22_H_30_O_14_ [M-H]^-^ 517.1563 517.1563 0.0 563.1563[M+HCOO]^-^ — — — — — SR

193.0535[ferulic acid-H]^-^

175.0374[feruloyl-H]^-^

103 13.25 Oleracein E C_12_H_13_NO_3_ [M-H]^-^ 218.0823 218.0823 0.0 — — — — — — MOC

[M+HCOO]^-^ 264.0871 264.0877 -2.3

[M+CH_3_COO]^-^ 278.1014 278.1034 -7.2

104 13.38 P-Methoxycinnamic acid C_10_H_10_O_3_ [M+HCOO]^-^ 223.0620 223.0612 3.6 — — — — — — SR

[M+CH_3_COO]^-^ 237.0750 237.0768 -7.6

105 13.55 Cassialoin C_21_H_22_O_9_ [M-H]^-^ 417.1232 417.1191 9.8 402.9965[M-H-CH_3_]^-^. [M+H]^+^ 419.1302 419.1337 -6.9 404.1066[M+H-CH_3_]^+^ RRR

[M+CH_3_COO]^-^ 477.1401 477.1402 -0.2 387.0458[M-H-2CH_3_]^-^ [M+Na]^+^ 441.1162 441.1156 1.4 389.0830[M+H-2CH_3_]^+^

357.0651[M-H-4CH_3_]^-^ 371.0750[M+H-2CH_3_-H_2_O]^+^

329.0441[M-H-4CH_3_-CO]^-^ 211.0292[M+H-2CH_3_-C_10_H_10_O_3_]^+^

106 13.65 Sibirioside A C_21_H_28_O_12_ [M-H]^-^ 471.1518 471.1508 2.1 323.0948[M-H-cinnamic acid [M+Na]^+^ 495.1462 495.1473 -4.7 — SR

[M+HCOO]^-^ 517.1545 517.1563 -3.5 -Glu]^-^

161.0410[Glu-H]^-^

107 13.90 2-[(R)-2-Hydroxypropyl]^-^5-

methyl-7-hydroxychromone C_13_H_12_O_4_ [M-H]^-^ 231.0641 231.0663 -9.5 — — — — — — RRR

[M+CH_3_COO]^-^ 291.0885 291.0874 3.8

108 13.98 1-O-galloyl-2-O-cinnamoyl-

β-D-glucose C_22_H_22_O_11_ [M-H]^-^ 461.1095 461.1089 1.3 285.0129[M-H-Glc]^-^ [M+H]^+^ 463.1228 463.1235 -1.5 — RRR

[M+CH_3_COO]^-^ 521.1262 521.1301 -7.5 315.0503[M-H-Rha]^-^

**Table S1. Characterization of the chemical constituents in KCSG by UHPLC–QTOF MS**

Peak t_R_ Identification Formula Negative mode Positive mode Source *^a^*

No (min) Quasi-molecular Observed Calculated ppm Fragement ions Quasi-molecular Observed Calculated ppm Fragement ions

ion mass (Da) mass (Da) ion mass (Da) mass (Da)

109 14.03 Levistilide A C_24_H_28_O_4_ [M-H]^-^ 379.1959 379.1952 1.8 — [M+H]^+^ 381.2050 381.2060 -2.6 191.1041[M+H-C_12_H_14_O_2_]^+^ ASR

[M+Na]^+^ 403.1884 403.1880 1.0 173.1047[M+H-C_12_H_14_O_2_-H_2_O]^+^

149.0609[M+H-C_12_H_14_O_2_-C_3_H_6_]^+^

135.0429[M+H-C_12_H_14_O_2_-C_3_H_6_-CH_2_]^+^

110 14.29 Eriodictyol C_15_H_12_O_6_ [M-H]^-^ 287.0557 287.0561 -1.4 — — — — — — AFI

[M+CH_3_COO]^-^ 347.0755 347.0772 -4.9

111 14.32 Salvianolic acid C C_26_H_20_O_10_ [M+HCOO]^-^ 537.1051 537.1038 2.4 473.0281[M-H-H_2_O]^-^ — — — — — SMR/CR

311.0217[M-H-C_9_H_8_O_2_]^-^

295.0609[M-H-C_9_H_8_O_5_]^-^

293.0480[M-H-C_9_H_10_O_5_]^-^

267.0535[M-H-CO-CO_2_]^-^

135.0476[M-H-C_18_H_12_O_8_]^-^

112 14.37 Resveratrol-4'-O-(6"- C_27_H_26_O_12_ [M-H]^-^ 541.1369 541.1351 3.3 — — — — — — RRR

galloyl) glucoside

113 14.39 (E)-3-hexenyl β-D-glucopyranoisde C_12_H_22_O_6_ [M-H]^-^ 261.1336 261.1344 -3.5 — — — — — — CR

114 14.45 Isonaringin or Naringin C_27_H_32_O_14_ [M-H]^-^ 579.1736 579.1719 2.9 459.1279[M-H-C_8_H_8_O]^-^ [M+H]^+^ 581.1859 581.1865 -1.0 383.1090[M+H-Rha-H_2_O]^+^ MOC/AFI

271.0617[M-H-Rha-Glc]^-^ [M+Na]^+^ 603.1696 603.1684 2.0 273.0697[M+H-Rha-Glc]^+^

151.0039[M-H-Rha-Glc-C_8_H_8_O]^-^ 153.0147[M+H-Rha-Glc-C_8_H_8_O]^+^

115 14.60 Hesperidin C_28_H_34_O_15_ [M-H]^-^ 609.1838 609.1825 2.1 301.0324[M-H-Rha-Glc]^-^ [M+H]^+^ 611.1948 611.1970 -3.6 465.1321[M+H-C_6_H_10_O_4_]^+^ MOC/AFI

286.0721[M-H-Rha-Glc-CH_3_]^-^ 449.1380[M+H-Rha]^+^

303.0800[M+H-Rha-Glc]^+^

153.0128[M+H-Rha-Glc-C_9_H_10_O_2_]^+^

116 14.82 Pratensein 7-O-Glucoside C_22_H_22_O_11_ [M-H]^-^ 461.1048 461.1089 -8.9 285.0249[M-H-Glc]^-^ — — — — — CR

[M+HCOO]^-^ 507.1140 507.1144 -0.8

117 15.03 Physcion 8-Gentiobioside C_28_H_32_O_15_ [M+HCOO]^-^ 653.1741 653.1723 2.8 299.0364[M-H-Rha-Glc]^-^ — — — — — RRR

284.0272[M-H-Rha-Glc-CH_3_]^-^

300.0631[M-H-3CH_3_-CO]^-^

285.0446[M-H-4CH_3_-CO]^-^

119 15.15 Azelaic acid C_9_H_16_O_4_ [M-H]^-^ 187.0975 187.0976 -0.5 169.0891[M-H-H_2_O]^-^ [M+Na]^+^ 211.0959 211.0941 8.5 — CR

143.1121[M-H-CO_2_]^-^

118 15.08 Methyl rosmarinate C_19_H_18_O_8_ [M+CH_3_COO]^-^ 433.1117 433.1140 -5.3 358.0873[M-H-CH_3_]^-^ [M+H]^+^ 375.1056 375.1074 -4.8 360.0790[M+H-CH_3_]^+^ SMR

343.0762[M-H-2CH_3_]^-^ 345.0753[M+H-2CH_3_]^+^

328.0604[M-H-3CH_3_]^-^ 327.0422[M+H-2CH_3_-H_2_O]^+^

125.0183[M-H-CO_2_-H_2_O]^-^

97.0249[M-H-CO_2_-H_2_O-C_2_H_4_]^-^

83.0133[M-H-CO_2_-H_2_O-C_2_H_4_-CH_2_]^-^

69.0321[M-H-CO_2_-H_2_O-C_2_H_4_-CH_2_-CH_2_]^-^

120 15.45 Formononetin C_16_H_12_O_4_ [M+HCOO]^-^ 313.0709 313.0718 -2.8 252.0567[M-CH_3_]^-^ [M+H]^+^ 269.0827 269.0808 7.1 254.1167[M+H-CH_3_]^+^ CR/ASR

237.1056[M+H-CH_3_OH]^+^

226.0059[M+H-CH_3_-CO]^+^

213.0228[M+H-CO-CO]^+^

197.0858[M+H-CH_3_-CO-CHO]^+^

163.0357[M+H-C_7_H_7_O]^+^

137.0593[M+H-C_7_H_7_O-C_2_H_2_]^+^

118.0638[M+H-C_7_H_7_O-CO-OH]^+^

121 15.52 (Z)-Muurola-4(14),5-Diene C_15_H_24_ — — — — — [M+H]^+^ 205.1963 205.1951 6.0 163.1038[M+H-C_3_H_6_]^+^ MOC

135.0759[M+H-C_3_H_6_-C_2_H_4_]^+^

121.0992[M+H-C_3_H_6_-C_2_H_4_-CH_2_]^+^

107.0784[M+H-C_3_H_6_-C_2_H_4_-CH_2_-CH_2_]^+^

122 15.73 2,5-dimethyl-7-hydroxychromone C_11_H_10_O_3_ [M-H]^-^ 189.0555 189.0557 -1.1 205.0696[M-H+O]^-^ — — — — — RRR

123 15.83 Myricetin C_15_H_10_O_8_ [M-H]^-^ 317.0328 317.0303 7.9 — — — — — — RRR

124 15.91 Osthole C_15_H_16_O_3_ [M+HCOO]^-^ 289.1095 289.1081 4.8 — — — — — — ASR

125 16.03 Naringenin 4'-O-Glucoside C_21_H_22_O_10_ [M+Cl]^-^ 469.0887 469.0907 -4.3 271.0607[M-H-Glc]^-^ [M+H]^+^ 435.1298 435.1286 2.8 273.0715[M+H-Glc]^+^ AFI

[M+CH_3_COO]^-^ 493.1370 493.1351 3.9 151.0028[M-H-Glc-C_8_H_6_O]^-^ 153.0136[M+H-Glc-C_8_H_6_O]^+^

126 16.19 Dicaffeoylquinic acid C_25_H_24_O_12_ [M-H]^-^ 515.1205 515.1195 1.9 353.0165[M-H-C_9_H_6_O_3_]^-^ — — — — — ASR/SR

191.0667[M-H-2C_9_H_6_O_3_]^-^

173.0947[M-H-2C_9_H_6_O_3_-H_2_O]^-^

127 16.28 Rhamnocitrin C_16_H_12_O_6_ [M+HCOO]^-^ 345.0644 345.0616 8.1 284.0841[M-H-CH_3_]^-^ [M+Na]^+^ 323.0539 323.0526 4.0 258.0985[M+H-CH_3_-CO]^+^ CR

[M+CH_3_COO]^-^ 359.0807 359.0772 9.8 229.0963[M+H-CH_3_-CO-CHO]^+^

**Table S1. Characterization of the chemical constituents in KCSG by UHPLC–QTOF MS**

Peak t_R_ Identification Formula Negative mode Positive mode Source *^a^*

No (min) Quasi-molecular Observed Calculated ppm Fragement ions Quasi-molecular Observed Calculated ppm Fragement ions

ion mass (Da) mass (Da) ion mass (Da) mass (Da)

128 16.28 Rosmarinic acid C_18_H_16_O_8_ [M-H]^-^ 359.0782 359.0772 2.8 197.0456[M-H-C_9_H_6_O_3_]^-^ [M+Na]^+^ 383.0748 383.0737 2.9 — SMR

179.0341[M-H-C_9_H_6_O_3_-H_2_O]^-^

161.0241[M-H-C_9_H_10_O_5_]^-^

129 16.28 Salvianolic acid F C_17_H_14_O_6_ [M-H]^-^ 313.0687 313.0718 -9.9 295.0440[M-H-H_2_O]^-^ — — — — — SMR

[M+HCOO]^-^ 359.0782 359.0772 2.8 269.0944[M-H-CO_2_]^-^

109.0230[M-H-C_11_H_8_O_4_]^-^

130 16.32 Senkyunolide H C_12_H_16_O_4_ — — — — — [M+H]^+^ 225.1138 225.1121 7.6 207.0697[M+H-H_2_O]^+^ ASR

[M+Na]^+^ 247.0928 247.0941 -5.3 189.1137[M+H-2H_2_O]^+^

165.0538[M+H-H_2_O-CO-CH_2_]^+^

131 16.97 Hesperetin 7-O-Glucoside C_22_H_24_O_11_ [M-H]^-^ 463.1240 463.1246 -1.3 — — — — — — AFI

132 17.05 Pilloin C_17_H_14_O_6_ [M+HCOO]^-^ 359.0752 359.0772 -5.6 285.0743[M-H-CO]^-^ — — — — — SMR

[M+CH_3_COO]^-^ 373.0935 373.0929 1.6 283.0839[M-H-CH_2_O]^-^

269.0349[M-H-C_2_H_3_O]^-^

133 17.11 Dibutyl succinate C_12_H_22_O_4_ [M-H]^-^ 229.1446 229.1445 0.4 — — — — — — RRR

134 17.11 Lithospermic acid C_27_H_22_O_12_ [M-H]^-^ 537.1048 537.1038 1.9 493.1129[M-H-COOH]^-^ — — — — — SMR

383.1017[M-H-C_7_H_6_O_4_]^-^

197.0534[M-H-C_18_H_12_O_7_]^-^

339.0679[M-H-C_9_H_10_O_5_]^-^

135 17.20 Nicotinic acid C_6_H_5_NO_2_ [M-H]^-^ 122.0237 122.0248 -9.0 — [M+H]^+^ 124.0382 124.0393 -8.9 96.0347[M+H-CO]^+^ RS

[M+HCOO]^-^ 168.0301 168.0302 -0.6 80.0488[M+H-CO_2_]^+^

78.0329[M+H-HCOOH]^+^

136 17.30 Estrone 3-Glucuronide C_24_H_30_O_8_ [M-H]^-^ 445.1824 445.1868 -9.9 253.0557[M-H-C_6_H_8_O_7_]^-^ — — — — — ASR

[M+HCOO]^-^ 491.1943 491.1923 4.1 192.1079[M-H-C_18_H_21_O]^-^

137 17.52 Hexanal C_6_H_12_O [M+HCOO]^-^ 145.0884 145.0870 9.6 — — — — — — ASR

[M+CH_3_COO]^-^ 159.1024 159.1027 -1.9

138 17.67 Angoroside C C_36_H_48_O_19_ [M-H]^-^ 783.2746 783.2717 3.7 607.2260[M-H-C_6_H_11_O_4_­­­-CHO]^-^ [M+Na]^+^ 807.2653 807.2682 -3.6 — SR

193.0497[M-H-C_26_H_38_O_15_]^-^

175.0398[M-H-C_26_H_38_O_15_-H_2_O]^-^

139 17.82 Lobetyolin C_20_H_28_O_8_ [M+HCOO]^-^ 441.1768 441.1766 0.5 341.0019[M-H-C_14_H_16_O_2_]^-^ — — — — — CR

140 17.85 Rosmarimic acid C_18_H_16_O_8_ [M-H]^-^ 359.0747 359.0772 -7.0 419.0387[M-H+CH_3_COOH]^-^ — — — — — CR

[M+HCOO]^-^ 405.0830 405.0827 0.7 135.0437[M-H-C_9_H_6_O_3_-H_2_O-CO_2_]^-^

141 17.88 Eriodictyol-7-glucoside C_21_H_22_O_11_ [M+HCOO]^-^ 495.1153 495.1144 1.8 — — — — — — MOC/AFI/RRR

142 18.04 Apigenin C_15_H_10_O_5_ [M-H]^-^ 269.0445 269.0455 -3.7 251.0619[M-H-H_2_O]^-^ — — — — — AFI

[M+HCOO]^-^ 326.1370 326.1398 -8.6 241.1352[M-H-CO]^-^

225.0479[M-H-CO_2_]^-^

223.0634[M-H-H_2_O-CO]^-^

195.0528[M-H-H_2_O-2CO]^-^

143 18.09 Meranzin hydrate C_15_H_18_O_5_ — — — — — [M+H]^+^ 279.1222 279.1227 -1.8 261.1080[M+H-H_2_O]^+^ AFI

243.0949[M+H-2H_2_O]^+^

189.0503[M+H-H_2_O-C_4_H_11_O_2_]^+^

103.0528[M+H-H_2_O-C_4_H_11_O-2O-CH_3_-2CO]^+^

144 18.11 2'-Deoxyinosine C_10_H_12_N_4_O_4_ [M+CH_3_COO]^-^ 311.1010 311.0997 4.2 — — — — — — ASR

145 18.60 Kaempferol 3-O-(6''-O-

acetyl)glucoside C_23_H_22_O_12_ [M-H]^-^ 489.1041 489.1038 0.6 313.0618[M-H-GluA]^-^ — — — — — RRR

298.0589[M-H-GluA-CH_3_]^-^

283.0443[M-H-GluA-2CH_3_]^-^

146 18.61 Formononetin-7-O-β-D-glucuronide C22H20O10 [M+HCOO]^-^ 489.1042 489.1038 0.8 — [M+H]^+^ 445.1118 445.1129 -2.5 269.0811[M+H-Glc] ^+^ ASR

147 19.02 Xanthotoxol C_11_H_6_O_4_ [M-H]^-^ 201.0181 201.0193 -6.0 — [M+H]^+^ 203.0331 203.0339 -3.9 175.0546[M+H-CO]^+^ ASR/AFI

147.0414[M+H-2CO]^+^

131.0477[M+H-CO-CO_2_]^+^

159.0695[M+H-CO_2_]^+^

91.0483[M+H-3CO-H_2_O]^+^

148 19.26 (-)-Epicatechin gallate C_22_H_18_O_10_ [M+HCOO]^-^ 487.0910 487.0882 5.8 — — — — — — RRR

[M+CH_3_COO]^-^ 501.1043 501.1038 1.0

149 19.49 Cinnamic acid C_9_H_8_O_2_ [M-H]^-^ 147.0448 147.0452 -2.7 103.0471[M-COOH]^-^ [M+H]^+^ 149.0583 149.0597 -9.4 131.0394[M+H-H_2_O]^+^ ASR/SR

103.0458[M+H-HCOOH]^+^

150 19.67 Citreorosein C_15_H_10_O_6_ [M-H]^-^ 285.0404 285.0405 -0.4 133.0168[M-C_7_H_4_O_4_]^-^ — — — — — RRR

93.0291[M-H-C_9_H_4_O_5_]^-^

**Table S1. Characterization of the chemical constituents in KCSG by UHPLC–QTOF MS**

Peak t_R_ Identification Formula Negative mode Positive mode Source *^a^*

No (min) Quasi-molecular Observed Calculated ppm Fragement ions Quasi-molecular Observed Calculated ppm Fragement ions

ion mass (Da) mass (Da) ion mass (Da) mass (Da)

151 20.20 Salvianolic acid B C_36_H_30_O_16_ [M-H]^-^ 717.1476 717.1461 2.1 519.0944[M-H-C_9_H_10_O_5_]^-^ [M+H]^+^ 719.1586 719.1607 -2.9 — CR

339.0545[M-H-C_9_H_10_O_5_-C_9_H_8_O_4_]^-^ [M+Na]^+^ 741.1391 741.1426 -4.7

321.0425[M-H-C_9_H_10_O_5_-C_9_H_10_O_5_]^-^

295.0524[M-H-C_9_H_10_O_5_- C_9_H_9_O_4_-CO_2_]^-^

152 20.38 Chrysophanol-O-glucoside C_21_H_20_O_9_ [M-H]^-^ 415.1001 415.1035 -8.2 — [M+Na]^+^ 439.0975 439.1000 -5.7 399.0175[M+H-H_2_O]^+^ MOC/RRR

[M+HCOO]^-^ 461.1060 461.1089 -6.3 381.1408[M+H-2H_2_O]^+^

363.8765[M+H-3H_2_O]^+^

351.0363[M+H-2H_2_O-CH_2_O]^+^

321.1612[M+H-2H_2_O-2CH_2_O]^+^

297.0232[M+H-C_4_H_8_O_4_]^+^

267.0948[M+H-C_4_H_8_O_4_-CH_2_O]^+^

153 20.42 Salvianolic acid A C_26_H_22_O_10_ [M-H]^-^ 493.1155 493.1140 3.0 383.0869[M-H-C_6_H_6_O_2_]^-^ [M+Na]^+^ 517.1111 517.1105 1.2 — CR/SMR

295.0703[M-H-C_9_H_10_O_5_]^-^

519.0837[M-H-C_9_H_10_O_5_]^-^

154 20.46 Kaempferol or luteolin C_15_H_10_O_6_ [M-H]^-^ 285.0382 285.0405 -8.1 151.0510[M-H-C_8_H_6_O_2_]^-^ — — — — — CR

133.0075[M-H-C_7_H_4_O_4_]^-^

107.0082[M-H-C_8_H_6_O_2_-CO_2_]^-^

155 30.80 Caffeic acid C_9_H_8_O_4_ — — — — — [M+Na]^+^ 203.0338 203.0338 0.0 163.0653[M+H-H_2_O]^+^ SMR/SR

135.0797[M+H-H_2_O-CO]^+^

89.0385[M+H-H_2_O-CO-HCOOH]^+^

156 20.99 Quercetin C_15_H_10_O_7_ [M-H]^-^ 301.0335 301.0354 -6.3 257.0919[M-H-CO_2_]^-^ — — — — — MOC/RRR

151.0065[M-H-C_8_H_6_O_3_]^-^

125.0228[M-H-C_9_H_4_O_4_]^-^

157 21.00 Didymin C_28_H_34_O_14_ [M-H]^-^ 593.1886 593.1876 1.7 285.0775[M-H-Rha-Glc]^-^ [M+H]^+^ 595.2002 595.2021 -3.2 433.1374[M+H-C_9_H_10_O-CO]^+^ AFI

[M+HCOO]^-^ 639.1924 639.1931 -1.1 241.0686[M-H-Rha-Glc-CO]^-^ [M+Na]^+^ 617.1808 617.1841 -5.4 287.0827[M+H-Rha-Glc]^+^

[M+CH_3_COO]^-^ 653.2041 653.2087 -7.0 151.0034[M-H-Rha-Glc-C_9_H_10_O]^-^ 153.0193[M+H-Rha-Glc-C_9_H_10_O-CO]^+^

158 21.03 Emodic acid C_15_H_8_O_7_ [M-H]^-^ 299.0196 299.0197 -0.3 271.0074[M-H-CO]^-^ — — — — — RRR

254.0209[M-H-COOH]^-^

159 21.23 Asimilobine C_17_H_17_NO_2_ [M+CH_3_COO]^-^ 326.1399 326.1398 0.3 — — — — — — MOC

160 21.23 Corytuberine C_19_H_21_NO_4_ [M-H]^-^ 326.1399 326.1398 0.3 — — — — — — MOC

161 21.23 Nornuciferine C_18_H_19_NO_2_ [M-H]^-^ 280.1365 280.1343 7.9 — — — — — — MOC

[M+HCOO]^-^ 326.1398 326.1398 0.0

162 21.43 Emodin 8-O-β-D-glucoside C_21_H_20_O_10_ [M-H]^-^ 431.0989 431.0984 1.2 269.0587[M-H-Glc]^-^ — — — — — CR/MOC/RRR

241.0580[M-H-Glc-CO]^-^

225.0917[M-H-Glc-CO_2_]^-^

181.0337[M-H-Glc-CO_2_-CH_3_-CO]^-^

163 21.48 Proline C_5_H_9_NO_2_ [M-H]^-^ 114.0558 114.0561 -2.6 — [M+H]^+^ 116.0709 116.0706 2.6 70.0650[M+H-HCOOH]^+^ RS/SR/ASR

68.0484[M+H-HCOOH-H_2_O]^+^

72.0795[M+H-CO_2_]^+^

164 21.49 Laccaic acid D C_16_H_10_O_7_ [M-H]^-^ 313.0355 313.0354 0.3 269.0554[M-H-CO_2_]^-^ [M+H]^+^ 315.0521 315.0499 7.0 300.1187[M+H-CH_3_]+ RRR

285.0667[M+H-2CH_3_]+

271.0877[M+H-CH_3_-CO]+

185.0627[M+H-C_9_H_6_O]+

165 21.52 Poncirin C28H_34_O_14_ [M-H]^-^ 593.1892 593.1876 2.7 575.1331[M-H-H_2_O]^-^ — — — — — AFI

[M+HCOO]^-^ 639.1889 639.1931 -6.6 285.0989[M-H-Rha-Glc]^-^

151.0474[M-H-Rha-Glc-C_9_H_10_O]^-^

166 21.93 8-O-Feruloylharpagide C_25_H_32_O_13_ [M-H]^-^ 539.1790 539.1770 3.7 345.1199[M-H-feruloyl-H_2_O]^-^. — — — — — SR

193.0356[ferulic acid-H]^-^

183.0663[harpagide-H- Glu -H_2_O]^-^

175.0395[feruloyl-H]^-^

167 21.93 Harpagoside C_24_H_30_O_11_ [M-H]^-^ 493.1727 493.1715 2.4 147.0495[M-H-C_15_H_22_O_9_]^-^ — — — — — SR

[M+HCOO]^-^ 539.1790 539.1770 3.7

168 21.93 Salidroside C_14_H_20_O_7_ [M-H]^-^ 299.1135 299.1136 -0.3 — — — — — — MOC

[M+HCOO]^-^ 345.1192 345.1191 0.3

169 22.01 Chrysophanol C_15_H_10_O_4_ [M-H]^-^ 253.0506 253.0506 0.0 225.0558[M-H-CO]^-^ [M+H]^+^ 255.0634 255.0652 -7.1 — MOC/RRR

210.0347[M-H-CO-CH_3_]^-^

182.0368[M-H-2CO-CH_3_]^-^

170 22.65 Ethyl hexanoate C_8_H_16_O_2_ [M+HCOO]^-^ 189.1118 189.1132 -7.4 — — — — — — MOC

[M+CH_3_COO]^-^ 203.1284 203.1289 -2.5

171 22.84 Naringenin C_15_H_12_O_5_ [M-H]^-^ 271.0614 271.0612 0.7 151.0013[M-H-C_8_H_8_O]^-^ [M+H]^+^ 273.0739 273.0757 -6.6 153.0141[M+H-C_8_H_8_O]^+^ MOC

107.0144[M-H-C_8_H_8_O-CO_2_]^-^ 91.0516[M+H-C_8_H_8_O-CO_2_-H_2_O]^+^

**Table S1. Characterization of the chemical constituents in KCSG by UHPLC–QTOF MS**

Peak t_R_ Identification Formula Negative mode Positive mode Source *^a^*

No (min) Quasi-molecular Observed Calculated ppm Fragement ions Quasi-molecular Observed Calculated ppm Fragement ions

ion mass (Da) mass (Da) ion mass (Da) mass (Da

172 22.96 Xanthotoxin C_12_H_8_O_4_ — — — — — [M+H]^+^ 217.0489 217.0495 -2.8 202.1728[M+H-CH_3_]^+^ ASR

161.0605[M+H-2CO]^+^

146.0545[M+H-2CO-CH_3_]^+^

174.0914[M+H-CH_3_-CO]^+^

118.0344[M+H-3CO-CH_3_]^+^

173 23.02 Magnolignan A C_18_H_20_O_4_ [M-H]^-^ 299.1287 299.1289 -0.7 239.0798[M-H-C_2_H_4_O_2_]^-^ [M+H]^+^ 301.1417 301.1434 -5.7 — MOC

267.1599[M-H-CH_2_O]^-^ [M+Na]^+^ 323.1285 323.1254 9.6

221.1170[M-H-C_2_H_4_O_2_-H_2_O]^-^

174 23.07 Salvianolic acid D C_20_H_18_O_10_ [M-H]^-^ 417.0833 417.0827 1.4 383.2236[M-H-C_7_H_6_O_4_]^-^ [M+H]^+^ 419.0998 419.0973 6.0 — SMR

197.0373[M-H-C_11_H_9_O_5_]^-^

[M+Na]^+^ 441.0777 441.0792 -3.4

175 23.88 Magnolol C_18_H_18_O_2_ [M-H]^-^ 265.1229 265.1234 -1.9 247.0740[M-H-H_2_O]^-^ — — — — — MOC

[M+HCOO]^-^ 311.1283 311.1289 -1.9 245.0546[M-H-H_2_O-2H]^-^

224.0842[M-H-C_3_H_5_]^-^

223.0804[M-H-C_3_H_6_]^-^

176 24.01 Thymidine C_10_H_14_N_2_O_5_ — — — — — [M+H]^+^ 243.0987 243.0975 4.9 — ASR

177 24.08 3,5,6,7,3',4'-Hexamethoxy C_21_H_22_O_8_ [M-H]^-^ 401.1238 401.1242 -1.0 — [M+H]^+^ 403.1353 403.1387 -5.5 373.1270[M+H-2CH_3_]^+^ AFI

flavone [M+Na]^+^ 425.1244 425.1207 8.7 165.0643[M+H-2CH_3_-C_10_H_10_-O_2_-CO-H_2_O]^+^

178 24.08 Coniferyl ferulate C_20_H_20_O_6_ [M+HCOO]^-^ 401.1238 401.1242 -1.0 178.0643[M-H-C_10_H_10_O_2_-CH_3_]^-^ — — — — — ASR

149.0616[M-H-C_10_H_10_O_2_-CO_2_]^-^

134.0408[M-H-C_10_H_10_O_2_-CH_3_-CO_2_]^-^

179 24.41 Costunolide C_15_H_20_O_2_ [M-H]^-^ 231.1389 231.1391 -0.8 — — — — — — AR

[M+HCOO]^-^ 277.1442 277.1445 -1.1

[M+CH_3_COO]^-^ 291.1681 291.1680 0.3

180 24.48 Physcion C_16_H_12_O_5_ [M-H]^-^ 283.0612 283.0612 0.0 268.0352[M-H-CH_3_]^-^ [M+H]^+^ 285.0743 285.0757 -4.9 270.0180[M+H-CH_3_]^+^ RRR

[M+CH_3_COO]^-^ 343.0814 343.0823 -2.6 240.0426[M-H-CH_3_-CO]^-^ 167.0552[M+H-C_8_H_6_O]^+^

212.0504[M-H-CH_3_-2CO]^-^ 139.8928[M+H-C_8_H_6_O-CO]^+^

184.0585[M-H-CH_3_-3CO]^-^ 123.1068[M+H-C_8_H_6_O-CO_2_]^+^

181 24.49 Beta-Lapachone C_15_H_14_O_3_ [M-H]^-^ 241.0868 241.0870 -0.8 223.0294[M-H-H_2_O]^-^ — — — — — SMR

197.1247[M-H-C_2_H_4_O]^-^

133.0169[M-H-C_6_H_4_O_2_]^-^

182 24.53 17-Hydroxycryptotanshinone C_19_H_20_O_4_ [M-H]^-^ 311.1264 311.1289 -8.0 295.0703[M-H -H_2_O]^-^ — — — — — SMR

[M+CH_3_COO]^-^ 371.1504 371.1500 1.9

183 24.95 Calycosin C_16_H_12_O_5_ [M-H]^-^ 283.0594 283.0612 -6.4 268.0343[M-H-CH_3_]^-^ — — — — — CR

[M+HCOO]^-^ 329.0677 329.0677 0.0

184 24.97 Nortanshinone C_17_H_12_O_4_ [M+Cl]^-^ 315.0454 315.0430 7.6 253.8577[M+H-CO]^+^ — — — — — SMR

[M+HCOO]^-^ 325.0690 325.0718 -8.6 225.1059[M+H-C_3_H_4_O]^+^

283.0026[M+H-CO]^+^

185 25.24 (Z)-Ligustilide C_13_H_18_O [M+HCOO]^-^ 235.1329 235.1340 -4.7 — — — — — — ASR

[M+CH_3_COO]^-^ 249.1494 249.1496 -0.8

186 25.77 Carnosol C_20_H_26_O_4_ [M+HCOO]^-^ 375.1818 375.1813 1.3 — — — — — — SMR

187 26.04 9,12,13-Trihydroxyoctadec- C_18_H_34_O_5_ [M-H]^-^ 329.2348 329.2333 4.6 229.1380[M-H-C_6_H_12_O]^-^ — — — — — CR

10-enoic acid 211.1305[M-H-C_6_H_12_O-H_2_O]^-^

188 26.07 Physcion 8-Glucoside C_22_H_22_O_10_ [M-H]^-^ 445.1143 445.1140 0.7 385.0093[M-H-60]^-^ [M+H]^+^ 447.1273 447.1286 -2.9 285.2041[M+H-C_6_H_10_O_5_]^+^ CR

355.1712[M-H-90]^-^ [M+Na]^+^ 469.1127 469.1105 4.7 270.0703[M+H-C_6_H_10_O_5_-CH_3_]^+^

325.0174[M-H-120]^-^ 242.1575[M+H-C_6_H_10_O_5_-CH_3_-CO]^+^

189 26.08 Senkyunolide G C_12_H_16_O_3_ [M-H]^-^ 207.1013 207.1027 -6.8 163.1003[M-H-CO_2_]^-^ — — — — — ASR

190 26.48 Calycosin-7-O-Beta-D-Glucoside C_22_H_22_O_10_ [M-H]^-^ 445.1098 445.1140 -9.4 385.0731[M-H-60]^-^ — — — — — ASR

[M+HCOO]^-^ 491.1158 491.1195 -7.5 355.0691[M-H-90]^-^

325.0592[M-H-120]^-^

191 26.63 Emodin C_15_H_10_O_5_ [M-H]^-^ 269.0453 269.0455 -0.7 241.0661[M-H-CO]^-^ [M+H]^+^ 271.0581 271.0601 -7.4 — CR

225.0711[M-H-CO_2_]^-^

197.0331[M-H-CO_2_-CO]^-^

181.0624[M-H-CO_2_-CO-CH_3_]^-^

192 26.76 Epi-Cryptoacetalide C_18_H_22_O_3_ [M-H]^-^ 285.1524 285.1496 9.8 255.0302[M-H-C_2_H_6_]^-^ — — — — — SMR

[M+HCOO]^-^ 331.1551 331.1551 0.0 257.0437[M-H-CO]^-^,

239.0864[M-H-CO-H_2_O]^-^

214.0779[M-H-CO-CO-CH_3_]^-^

193 26.78 Marmin C_19_H_24_O_5_ [M-H]^-^ 331.1551 331.1551 0.0 — — — — — — AFI

**Table S1. Characterization of the chemical constituents in KCSG by UHPLC–QTOF MS**

Peak t_R_ Identification Formula Negative mode Positive mode Source *^a^*

No (min) Quasi-molecular Observed Calculated ppm Fragement ions Quasi-molecular Observed Calculated ppm Fragement ions

ion mass (Da) mass (Da) ion mass (Da) mass (Da)

194 26.89 Meranzin C_15_H_16_O_4_ [M-H]^-^ 259.0953 259.0976 -8.9 — — — — — — AFI

[M+CH_3_COO]^-^ 319.1182 319.1187 -1.6

195 27.14 2-Cyclohexylethanol C_8_H_16_O [M+HCOO]^-^ 173.1181 173.1183 -1.2 — — — — — — RS

[M+CH_3_COO]^-^ 187.1331 187.1340 -4.8

196 27.14 Nonanal C_9_H_18_O [M+HCOO]^-^ 187.1331 187.1340 -4.8 — — — — — — RS

197 27.14 Trans-Sabinol C_10_H_16_O [M+HCOO]^-^ 197.1197 197.1183 7.1 — — — — — — ASR

[M+CH_3_COO]^-^ 211.1336 211.1340 -1.9

198 27.18 Magnolignan E C_18_H_18_O_4_ [M-H]^-^ 297.1105 297.1132 -9.1 267.0721[M-H-CH_2_O]^-^ — — — — — MOC

[M+CH_3_COO]^-^ 357.1343 357.1344 -0.3 249.2223[M-H-CH_2_O-H_2_O]^-^

223.0722[M-H-H_2_O]^-^

199 27.21 5,7-Dihydroxy-3',4',5'- C_18_H_16_O_7_ [M-H]^-^ 343.0814 343.0823 -2.6 328.0622[M-H-CH_3_]^-^ [M+H]^+^ 345.0946 345.0969 -6.7 330.0680[M+H-CH_3_]^+^ AFI

Trimethoxyflavone 313.0383[M-H-2CH_3_]^-^ 315.0283[M+H-2CH_3_]^+^

298.0118[M-H-3CH_3_]^-^

270.0287[M-H-3CH_3_-CO]^-^

200 27.34 Limonin C_26_H_30_O_8_ [M-H]^-^ 469.1867 469.1868 -0.2 — [M+H]^+^ 471.1981 471.2013 -6.8 425.1919[M+H-CH_2_O_2_]^+^ AFI

[M+HCOO]^-^ 515.1936 515.1923 2.5 [M+Na]^+^ 493.1871 493.1833 7.7 339.1860[M+H-CH_2_O_2_-2CH_3_-H_2_O-CO]^+^

201 28.71 TanshinoneⅡA C_19_H_18_O_3_ [M-H]^-^ 293.1174 293.1183 -3.1 — [M+H]^+^ 295.1306 295.1329 -7.8 277.1215[M+H-H_2_O]^+^ SMR

[M+Na]^+^ 317.1346 317.1329 5.4 249.1257[M+H-H_2_O-CO]^+^

234.1017[M+H-H_2_O-CO-CH_3_]^+^

221.1263[M+H_2_O-CO-CO]^+^

206.1065[M+H-H_2_O-CO-CH_3_-CO]^+^

191.0832[M+H-H_2_O-CO-CH_3_-CO-CH_3_]^+^

280.1083[M+H-CH_3_]+ 266.0918[M+H-CHO]+

262.0968[M+H-H_2_O-CH_3_]+

202 27.37 Anthranone C_14_H_10_O [M+CH_3_COO]^-^ 253.0870 253.0870 0.0 — — — — — — RRR

203 27.37 Magnaldehyde E C_16_H_14_O_3_ [M-H]^-^ 253.0879 253.0870 3.6 184.0518[M-H-CO-C_3_H_5_]^-^ [M+Na]^+^ 277.0839 277.0835 1.4 — MOC

280.4818[M-H-CH_3_O]^-^

204 27.532 Rhein C_15_H_8_O_6_ [M-H]^-^ 283.0259 283.0248 3.9 239.0365[M-H-CO_2_]^-^ [M+H]+ 285.0363 285.0346 6.0 257.1623[M+H-CO]+ MOC/RRR

211.0441[M-H-CO_2_-CO]^-^ 267.1632[M+H-H_2_O]+

183.0450[M-H-CO_2_-CO-CO]^-^ 241.0366[M+H-CO_2_]+

183.0300[M-H-CO-C_3_H_6_]^-^

205 27.84 11-O-Acetyl-Aloe-Emodin C_17_H_12_O_6_ [M-H]- 311.0557 311.0561 -1.3 — — — — — — RRR

206 27.97 Xanthoplanine C_21_H_26_NO_4_ [M+HCOO]^-^ 401.1861 401.1844 4.2 313.1313[M-H-CO_2_]^-^ — — — — — MOC

207 27.97 Tiglylcarnitine C_12_H_21_NO_4_ [M-H]^-^ 242.1390 242.1398 -3.3 — — — — — — RRR

[M+HCOO]^-^ 288.1460 288.1453 2.4

208 28.18 Ethyl cholate C_26_H_44_O_5_ [M-H]^-^ 435.3113 435.3116 -0.7 — [M+H]^+^ 437.3259 437.3262 -0.7 — RRR

[M+CH_3_COO]^-^ 495.3349 495.3327 4.4 [M+Na]^+^ 459.3103 459.3081 4.8

209 28.39 Crichetocholic acid C_24_H_40_O_5_ [M-H]^-^ 407.2795 407.2803 -2.0 — — — — — — ASR

210 28.70 Dibutyl phthalate C_16_H_22_O_4_ [M-H]^-^ 277.1436 277.1445 -3.3 — [M+H]^+^ 279.1568 279.1591 -8.2 — ASR

211 28.71 1,6-dimethyl-1,2,8,9- C_18_H_16_O_3_ [M-H]^-^ 279.1025 279.1027 -0.7 261.0917[M-H-H_2_O]^-^ [M+H]^+^ 281.1173 281.1172 0.4 — SMR

tetrahydronaphtho[1,2-g] [M+HCOO]^-^ 325.1081 325.1081 0.0 233.0966[M-H-H_2_O-CO]^-^

[1]benzofuran-10,11-dione

212 28.73 N-Pentylbenzene C_11_H_16_ [M+CH_3_COO]^-^ 207.1385 207.1391 -2.9 — [M+Na]^+^ 441.1058 441.1050 1.8 — ASR

213 28.90 Randainal C_18_H_16_O_3_ [M-H]^-^ 279.1010 279.1027 -6.1 261.0013[M-H-H_2_O]^-^ — — — — — MOC

[M+HCOO]^-^ 325.1059 325.1081 -6.8 233.1379[M-H-H_2_O-CO]^-^

152.0414[M-H-C_9_H_9_]^-^

214 29.18 Gancaonin B C_21_H_20_O_6_ [M-H]^-^ 367.1200 367.1187 3.5 217.0820[M-H-C_9_H_10_O_2_]^-^ — — — — — SR

173.0284[M-H-C_9_H_10_O_2_-CO_2_]^-^

149.0266[M-H-C_12_H_10_O_4_]^-^

134.0471[M-H-C_12_H_10_O_4_-CH_3_]^-^

215 29.50 Methyl linoleate C_19_H_34_O_2_ [M+Cl]^-^ 329.2251 329.2253 -0.6 — — — — — — MOC

216 29.76 4′,5,6,7,8‐pentamethoxy C_20_H_20_O_7_ — — — — — [M+H]^+^ 373.1254 373.1282 -7.5 358.1023[M+H-CH_3_]^+^ AFI

‐flavone [M+Na]^+^ 395.1120 395.1101 4.8 181.0095[M+H-C_10_H_10_O_22_-CH_3_]^+^

153.0160[M+H-C_10_H_10_O_22_-CH_3_-CO]^+^

217 30.41 Stilbene C_14_H_12_ [M+HCOO]^-^ 225.0922 225.0921 0.4 — — — — — — RRR

[M+CH_3_COO]^-^ 239.1067 239.1078 -4.6

218 30.81 Dibutyl itaconate C_13_H_22_O_4_ [M-H]^-^ 241.1442 241.1445 -1.2 — — — — — — RRR

219 30.81 Bergamiol C_12_H_20_O_2_ [M+HCOO]^-^ 241.1442 241.1445 -1.2 — — — — — — ASR

220 30.87 N-Acetylnorsynephrine C_10_H_13_NO_3_ [M-H]^-^ 194.0822 194.0823 -0.5 165.0567[M-H-CHO]^-^ [M+Na]^+^ 218.0775 218.0788 -6.0 — AFI

224 31.12 Methyl (Z)-hexadec-2-enoate C_17_H_32_O_2_ [M+HCOO]^-^ 313.2388 313.2384 1.3 — — — — — — RS

**Table S1. Characterization of the chemical constituents in KCSG by UHPLC–QTOF MS**

Peak t_R_ Identification Formula Negative mode Positive mode Source *^a^*

No (min) Quasi-molecular Observed Calculated ppm Fragement ions Quasi-molecular Observed Calculated ppm Fragement ions

ion mass (Da) mass (Da) ion mass (Da) mass (Da)

225 31.37 1,2-Dihydrotanshinone I C_18_H_14_O_3_ — — — — — [M+H]^+^ 279.0991 279.1016 -9.0 261.0903[M+H-H_2_O]^+^ SMR

[M+Na]^+^ 301.0839 301.0835 1.3 233.0954[M+H-H_2_O-CO]^+^

264.1919[M+H-CH_3_]^+^

205.0974[M+H-C_2_H_2_O_3_]^+^

251.0956[M+H-CO]^+^ 246.0599[M+H-CH_3_-H_2_O]^+^

221 30.96 Pentadecanoic acid C_15_H_30_O_2_ [M+HCOO]^-^ 287.2225 287.2228 -1.0 — — — — — — ASR

222 30.99 Tormentic acid C_30_H_48_O_5_ [M-H]^-^ 487.3431 487.3429 0.4 — — — — — — SMR

223 31.06 Aloe-emodin C_15_H_10_O_5_ [M-H]^-^ 269.0462 269.0455 2.6 211.0846[M-H-2CHO]^-^ — — — — — MOC/RRR

167.0685[M-H-2CHO-CO_2_]^-^

240.0210[M-H-CHO]^-^

224 31.12 Methyl (Z)-hexadec-2-enoate C_17_H_32_O_2_ [M+HCOO]^-^ 313.2388 313.2384 1.3 — — — — — — RS

225 31.37 Methylenetanshinquinone C_18_H_14_O_3_ — — — — — [M+H]^+^ 279.0991 279.1016 -9.0 261.0896[M+H-H_2_O]^+^ SMR

[M+Na]^+^ 301.0839 301.0835 1.3 233.0947[M+H-H_2_O-CO]^+^

205.0994[M+H-H_2_O-CO-CO]^+^

226 31.75 4-Methylenemiltirone C_18_H_18_O_2_ [M-H]^-^ 265.1249 265.1234 4.9 224.0842[M-H-C_3_H_5_]^-^ [M+H]^+^ 267.1301 267.1380 3.0 — SMR

223.0804[M-H-C_3_H_6_]^-^

197.0609[M-H-C_3_H_4_-CO]^-^

196.0865[M-H-C_3_H_5_-CO]^-^

228 32.23 Butylphthalide C_12_H_14_O_2_ — — — — — [M+H]^+^ 191.1061 191.1067 -3.1 173.0937[M+H-H_2_O]^+^ ASR

145.0996[M+H-H_2_O-CO]^+^

117.0627[M+H-H_2_O-2CO]^+^

227 32.16 Heptadecanoic acid C_17_H_34_O_2_ [M+HCOO]^-^ 315.2539 315.2541 -0.6 — [M+Na]^+^ 293.2473 293.2451 7.5 — ASR

229 32.25 6-Undecanone C_11_H_22_O [M+HCOO]^-^ 215.1654 215.1653 0.5 — — — — — — ASR

230 32.29 Dehydrocostuslactone C_15_H_18_O_2_ [M-H]^-^ 229.1234 229.1234 0.0 — [M+H]^+^ 231.1381 231.1380 0.4 213.5393[M+H-H_2_O]^+^ AR

185.1555[M+H-CH_2_O_2_]^+^

157.0995[M+H-C_3_H_6_O_2_]^+^

231 32.29 Neocryptotanshinone C_19_H_22_O_4_ [M-H]^-^ 313.1446 313.1445 0.3 269.1509[M-H-CO_2_]^-^ [M+H]^+^ 315.1568 315.1591 -7.3 279.1370[M+H-2H_2_O]^+^ SMR

[M+Na]^+^ 337.1441 337.1410 9.2 163.0430[M+H-C_10_H_8_O]^+^

107.0738[M+H-C_10_H_8_O-2CO] ^+^

232 32.73 Hexadecanoic acid or Palmitic acid C_16_H_32_O_2_ [M+CH_3_COO]^-^ 315.2541 315.2541 0.0 — [M+H]^+^ 257.2491 257.2475 6.2 — RS/RRR/ASR

[M+Na]^+^ 279.2301 279.2295 2.2

233 33.01 Obovatol C_18_H_18_O_3_ [M-H]^-^ 281.1191 281.1183 2.9 240.0833[M-H-C_3_H_5_]^-^ [M+H]^+^ 283.1335 283.1329 2.1 — MOC

164.0478[M-H-C_9_H_9_]^-^

133.0661[M-H-C_9_H_8_O_2_]^-^

234 33.13 Methyl palmitate C_17_H_34_O_2_ [M+HCOO]^-^ 315.2520 315.2541 -6.7 — — — — — — MOC/RS

235 33.45 LysoPE(0:0/16:0) C_21_H_44_NO_7_P [M-H]^-^ 452.2780 452.2783 -0.7 — — — — — — RRR

236 33.87 Cryptotanshinone C_19_H_20_O_3_ [M-H]^-^ 295.1354 295.1340 4.7 238.0895[M-H-CO_2_-CH]^-^ [M+H]^+^ 297.1467 297.1485 -6.1 279.2296[M+H-H_2_O]^+^ SMR

[M+CH_3_COO]^-^ 355.1563 355.1551 3.4 253.1583[M+H-CO_2_]^+^

251.0690[M+H-H_2_O-CO]^+^

237 34.22 Alpha-Linolenic Acid C_18_H_30_O_2_ [M-H]^-^ 277.2176 277.2173 1.1 — [M+H]^+^ 279.2305 279.2319 -5.0 — RS

[M+Na]^+^ 301.2148 301.2138 3.3

238 34.32 Coronaric acid C_18_H_32_O_3_ [M-H]^-^ 295.2278 295.2279 -0.3 277.2183[M-H-H_2_O]^-^ [M+H]^+^ 297.2401 297.2424 -7.7 — CR

251.2438[M-H-CO_2_]^-^

239 34.32 Sugiol C_20_H_28_O_2_ [M-H]^-^ 299.2018 299.2017 0.3 — [M+H]^+^ 301.2161 301.2162 -1.7 — SR

240 34.41 2-Hydroxymyristic acid C_14_H_28_O_3_ [M-H]^-^ 243.1960 243.1966 -2.5 — — — — — — RRR

241 36.61 1,2-Didehydrocryptotanshinone C_19_H_18_O_3_ [M-H]^-^ 293.1170 293.1183 -4.4 — [M+H]^+^ 295.1312 295.1329 -5.8 277.1215[M+H-H_2_O]^+^ SMR

[M+Cl]^-^ 331.0182 293.1183 -4.4 [M+Na]^+^ 317.1154 317.1148 1.9 249.1257[M+H-H_2_O-CO]^+^

[M+HCOO]^-^ 339.1231 339.1238 -2.1 221.1263[M+H-H_2_O-CO-CO]^+^

242 39.13 Ethylcyclohexane C_8_H_16_ — — — — — [M+H]^+^ 113.1319 113.1325 -5.3 — RS

243 40.52 Synephrine C_9_H_13_NO_2_ — — — — — [M+H]^+^ 168.1013 168.1019 -3.6 — AFI

[M+Na]^+^ 190.0834 190.0838 -2.1

244 41.32 Oleic acid C_18_H_34_O_2_ [M-H]^-^ 281.2488 281.2486 0.7 267.3661[M-CH_3_]^-^ [M+H]^+^ 283.2617 283.2632 -5.3 — RRR/ASR

245 41.75 L-Palmitoylcarnitine C_23_H_45_NO_4_ [M-H]^-^ 398.3272 398.3276 -1.0 — — — — — — RRR

[M+HCOO]^-^ 444.3299 444.3331 -7.2

246 42.63 Stearic acid or Octadecanoic acid C18H36O2 [M-H]- 283.2640 283.2643 -1.1 — — — — — — ASR/RS

*^a^* RRR, *Rhei radix et rhizome*; MOC, *Magnolia officinalis cortex*; RS, *Raphani semen*; AFI, *Aurantii fructus immaturus*; CR, *Codonopsis radix*; SR, *Scrophulariae radix*; PS, *Persicae semen*; ASR, *Angelicae sinensis radix*; AR, *Aucklandiae radix*; SMR, *Salviae miltiorrhizae radix et rhizoma*.
