# Supplementary material for: Exploring the chemical components of Kuanchang-Shu granule and its protective effects of postoperative ileus in rats by regulating AKT/HSP90AA1/eNOS pathway
Source: Chin Med. 2024 Feb 21;19:29. doi: 10.1186/s13020-024-00892-3 (PMC10880223; doi:10.1186/s13020-024-00892-3)
Supplement: Supplementary file 2 — Additional file 2. The code of active ingredients of KCSG. [file 13020_2024_892_MOESM2_ESM.docx]

Additional file

**Table S2.** The code of active ingredients of KCSG.

**NO. Identification Code NO. Identification Code**

1 Trimethoxyflavone M1 31 Ferulic acid M31

2 Trans-Sabinol M2 32 Estrone 3-Glucuronide M32

3 TanshinoneⅡA M3 33 Eriodictyol M33

4 Tanshinone I M4 34 Epi-Cryptoacetalide M34

5 Synephrine M5 35 Emodin M35

6 Sugiol M6 36 Emodic acid M36

7 Sinapine thiocyanate M7 37 Dehydrocostuslactone M37

8 Sinapine M8 38 Cryptotanshinone M38

9 Rhein M9 39 Crichetocholic acid M39

10 Pipradrol M10 40 Costunolide M40

11 P-hydroxybenzoic acid-o-galloyl-glucoside M11 41 Corytuberine M41

12 Oleracein E M12 42 Chrysophanol M42

13 Obovatol M13 43 Calycosin M43

14 Nortanshinone M14 44 Butylphthalide M44

15 Neocryptotanshinone M15 45 Butanoic acid M45

16 Naringenin M16 46 Bergamiol M46

17 Marmin M17 47 Anthranone M47

18 Magnolol M18 48 Amygdalin M48

19 Magnolignan E M19 49 Aloe-emodin M49

20 Magnolignan A M20 50 5-HMF M50

21 Magnaldehyde E M21 51 5,7-Dihydroxy-3',4',5'-Trimethoxyflavone M51

22 Luteolin M22 52 4-Methylenemiltirone M52

23 Ketoleucine M23 53 3-Ethenyl-alpha-hydroxybenzeneacetic acid M53

24 Kaempferol M24 54 3,5-dihydroxybenzoic acid M54

25 Indole M25 55 2,5-dimethyl-7-hydroxychromone M55

26 Imperatorin M26 56 17-hydroxypregnenolone sulfate M56

27 Honokiol M27 57 11-O-Acetyl-Aloe-Emodin M57

28 Hesperitin M28 58 1,2-Dihydrotanshinone I M58

29 Gancaonin B M29 59 1,2-Didehydrocryptotanshinone M59

30 Formononetin M30 60 (Z)-Ligustilide M60
