# Supplementary material for: Exploring the chemical components of Kuanchang-Shu granule and its protective effects of postoperative ileus in rats by regulating AKT/HSP90AA1/eNOS pathway
Source: Chin Med. 2024 Feb 21;19:29. doi: 10.1186/s13020-024-00892-3 (PMC10880223; doi:10.1186/s13020-024-00892-3)
Supplement: Supplementary file 3 — Additional file 3. The relationship between the key active ingredients of KCSG and core targets of key active ingredients. [file 13020_2024_892_MOESM3_ESM.docx]

Additional file

# Additional Tables

**Table S3.** The relationship between the key active ingredients of KCSG and core targets of key active ingredients.

**Key Active Ingredient Classification Source Relevant Core Target**

M1 Flavonoids AFI MMP9, NOS2, NOS3, PTGS2

M2 Terpenoids ASR HMOX1

M3 Quinones SMR BCL2, ICAM1, JUN, MMP9, PTGS2, PTGS2

M4 Quinones SMR HSP90AA1, PTGS2

M6 Terpenoids SR PTGS2

M7 Alkaloids RS EGFR, HSP90AA1, MMP9, PTGS2

M9 Flavonoids MOC, RRR BCL2, JUN, PTGS2

M13 Other MOC NOS2, PTGS2

M15 Quinones SMR PTGS2, STAT3

M17 Coumarins AFI PTGS2

M18 Other MOC HSP90AA1, PTGS2

M19 Phenols MOC MAPK1

M20 Other MOC EGFR, MMP9

M21 Phenols MOC HSP90AA1

M22 Flavonoids CR AKT1, HMOX1, ICAM1, IFNG, IL10, IL2, IL4, IL6, JUN, MAPK1, MMP9, TNF, VEGFA, PTGS2

M24 Flavonoids CR AKT1, BCL2, HMOX1, ICAM1, JUN, NOS2, NOS3, STAT1, TNF, PTGS2

M25 Alkaloids MOC HSPA8

M26 Flavonoids AFI, ASR PTGS2

M27 Phenols MOC EGFR, HSP90AA1, PTGS2

M29 Flavonoids SR IL2, NOS2, PTGS2

M30 Flavonoids ASR, CR IL2, JUN, NOS2, NOS3, PTGS2

M31 Other ASR, SMR, SR HSP90AA1, MMP9, NOS3, PTGS2, STAT3

M33 Flavonoids AFI HMOX1, PTGS2

M34 Other SMR HMOX1, SRC

M35 Quinones CR BCL2, EGFR, HSP90AA1, IL1B, NOS2, TNF, PTGS2

M36 Flavonoids RRR EGFR, HSP90AA1, IL2, MMP9

M37 Terpenoids AR PTGS2

M38 Quinones SMRR TNF, PTGS2, STAT3

M40 Terpenoids AR IL1B, PTGS2

M42 Quinones MOC, RRR HSP90AA1, MMP9, PTGS2

M43 Flavonoids CR EGFR, NOS2, PTGS2

M44 Coumarins ASR ICAM1

M47 Other RRR BCL2

M48 Alkaloids PS HSP90AA1, VEGFA, PTGS2, PTGS2

M49 Quinones MOC, RRR IL1B, MMP9, TNF, PTGS2

M50 Other SR HSP90AA1, PTGS2

M51 Flavonoids AFI MMP9

M52 Quinones SMR NOS3, PTGS2

M55 Quinones RRR PTGS2

M57 Flavonoids RRR MMP9

M59 Quinones SMR STAT3
