# Supplementary material for: Exploring the chemical components of Kuanchang-Shu granule and its protective effects of postoperative ileus in rats by regulating AKT/HSP90AA1/eNOS pathway
Source: Chin Med. 2024 Feb 21;19:29. doi: 10.1186/s13020-024-00892-3 (PMC10880223; doi:10.1186/s13020-024-00892-3)
Supplement: Supplementary file 4 — Additional file 4. The relationship between pathways and the core targets with the active ingredients of KCSG. [file 13020_2024_892_MOESM4_ESM.docx]

***Additional file***

**Table S4.** The relationship between pathways and the core targets with the active ingredients of KCSG.

**Relevant Pathways Core Target**

HIF-1 signaling pathway IL6, IFNG, NOS2, NOS3, STAT3, BCL2, HMOX1, MAPK1, AKT1, TLR4, EGFR, VEGFA

C-type lectin receptor signaling pathway IL10, IL6, JUN, STAT1, SRC, IL1B, MAPK1, AKT1, PTGS2, TNF, IL2

IL-17 signaling pathway IL4, IL6, HSP90AA1, JUN, IFNG, IL1B, MAPK1, PTGS2, TNF, MMP9

JAK-STAT signaling pathway IL10, IL4, IL6, IFNG, STAT1, STAT3, BCL2, AKT1, EGFR, IL2

TNF signaling pathway IL6, JUN, IL1B, MAPK1, AKT1, PTGS2, TNF, MMP9, ICAM1

T cell receptor signaling pathway IL10, IL4, JUN, IFNG, MAPK1, AKT1, TNF, IL2

Toll-like receptor signaling pathway IL6, JUN, STAT1, IL1B, MAPK1, AKT1, TNF, TLR4

NOD-like receptor signaling pathway IL6, HSP90AA1, JUN, STAT1, IL1B, BCL2, MAPK1, TNF, TLR4

PI3K-Akt signaling pathway IL4, IL6, HSP90AA1, NOS3, BCL2, MAPK1, AKT1, TLR4, EGFR, IL2, VEGFA

Apoptosis ERN1, JUN, BCL2, BAX, MAPK1, BIRC5, AKT1, TNF

VEGF signaling pathway SRC, NOS3, MAPK1, AKT1, PTGS2, VEGFA

NF-kappa B signaling pathway IL1B, BCL2, PTGS2, TNF, TLR4, ICAM1

MAPK signaling pathway HSPA8, JUN, IL1B, MAPK1, AKT1, TNF, EGFR, VEGFA

FoxO signaling pathway IL10, IL6, STAT3, MAPK1, AKT1, EGFR

ErbB signaling pathway JUN, SRC, MAPK1, AKT1, EGFR
